# *Histoplasma* seropositivity and environmental risk factors for exposure in a general population in Upper River Region, The Gambia: A cross-sectional study

**DOI:** 10.1016/j.onehlt.2024.100717

**Published:** 2024-03-27

**Authors:** Tessa Rose Cornell, Bakary Conteh, Lamin Drammeh, Foday Jeffang, Ebrima Sallah, Alkali Kijera, Mustapha Jarju, Mehrab Karim, Ebrima Khan, Pa Ousman Ceesay, Ebrima Manneh, Regional Health Directorate Upper River Region, Dan G. Wootton, Gina Pinchbeck, Claire Elizabeth Scantlebury

**Affiliations:** a Institute of Infection, Veterinary and Ecological Sciences (IVES), University of Liverpool, UK; b Medical Research Council Unit The Gambia at the London School of Hygiene and Tropical Medicine, Fajara, The Gambia; c Gambia Bureau of Statistics (GBoS), Ministry of Finance and Economic Affairs, The Gambia; d Regional Health Directorate Upper River Region, Ministry of Health, The Gambia; e NIHR Health Protection Research Unit in Emerging & Zoonotic Diseases, University of Liverpool, UK

**Keywords:** Histoplasma, Seroprevalence, Human-animal-environment interface, One health, The Gambia

## Abstract

Robust surveillance of *Histoplasma* species is warranted in endemic regions, including investigation of community-level transmission dynamics. This cross-sectional study explored anti-*Histoplasma* antibody seroprevalence and risk factors for exposure in a general population in Upper River Region (URR), The Gambia.

Study participants were recruited (December 2022–March 2023) by random household sampling across 12 Enumeration Areas (EAs) of URR. A questionnaire and clinical examination were performed; exploring demographic, clinical and environmental risk factors for *Histoplasma* exposure. One venous blood sample per participant was subject to IMMY Latex Agglutination *Histoplasma* test to determine presence of a recent IgM response to *Histoplasma*. Seropositivity risk factors were explored by multi-level, multivariable logistic regression analysis.

The study population (*n* = 298) aged 5–83 years, demonstrated a positively skewed age distribution and comprised 55.4% females. An apparent seroprevalence of 18.8% (*n* = 56/298, 95% CI 14.5–23.7%) was measured using the LAT. A multivariable model demonstrated increased odds of *Histoplasma* seropositivity amongst female participants (OR = 2.41 95% CI 1.14–5.10); and participants reporting involvement in animal manure management (OR = 4.21 95% CI 1.38–12.90), and management of domestic animals *inside* the compound at night during the dry season (OR = 10.72 95% CI 2.02–56.83). Increasing age (OR = 0.96 95% CI 0.93–0.98) was associated with decreased odds of seropositivity. Clustering at EA level was responsible for 17.2% of seropositivity variance.

The study indicates frequent recent *Histoplasma* exposure and presents plausible demographic and environmental risk factors for seropositivity. *Histoplasma* spp. characterisation at this human-animal-environment interface is warranted, to determine public health implications of environmental reservoirs in The Gambia.

The study was supported by Wellcome Trust (206,638/Z/17/Z to CES) and a University of Liverpool-funded PhD studentship (to TRC).

## Highlights

• Presents evidence of *Histoplasma* seropositivity in a general population in The Gambia.
• Associations measured between seropositivity and participant gender and age.
• Seropositivity associated with domestic animal and livestock management activities.
• A One Health approach is warranted to examine *Histoplasma* epidemiology.

## 1 Introduction

*Histoplasma* species are globally distributed, thermally dimorphic fungi. Recent designation as high priority fungal pathogens by the World Health Organization highlighted the need for robust surveillance in endemic regions [[1], [2], [3]]. In low- and middle-income countries, the emerging global health threat of invasive fungal infections including histoplasmosis, is compounded by limitations to diagnostic and antifungal treatment access and affordability.

Disseminated histoplasmosis is a major defining disease amongst patients with advanced HIV disease (AHD) [[4], [5], [6]]. Although disease course in immunocompetent hosts is typically asymptomatic and self-limiting [7], acute histoplasmosis in individuals without co-morbidities has been reported [[8], [9], [10]]. A range of clinical presentations are described, including respiratory disease [11,12], which can mimic the clinical presentation and radiographic pattern of pulmonary Tuberculosis (TB) [13,14]; and cutaneous and bone lesions [[15], [16], [17]], described by the majority of case reports in West Africa [3].

*Histoplasma*-focussed research to date has concentrated on the following areas: (i) observational studies exploring the burden of *Histoplasma* infection in patient groups with defined HIV or TB infection status, and the demographic and clinical risk factors for infection [4,5,14,18,19]; (ii) case or outbreak reports, which allude to sources of exposure based on evidence of *Histoplasma* isolation from environmental reservoirs [12,20]; and (iii) phylogenetic comparison of *Histoplasma* isolates using evolving genomic techniques, to present the molecular epidemiology of *Histoplasma* spp. [21,22]. An evidence gap exists as to the burden of *Histoplasma* and the role of fungal reservoirs in the West African community context [23]. In rural The Gambia, farming communities living in close proximity to domestic animals represent a potentially susceptible population to exposure. This study examined *Histoplasma* through a One Health lens, by exploring environmental risk factors for exposure in a general population in Upper River Region (URR), The Gambia. *Histoplasma* is not a proven zoonosis; however, increased awareness of environmental risk factors might inform public health measures to reduce exposure in the community setting and ultimately, reduce the burden of fungal infections in patient populations in the clinical setting.

This study determined the seroprevalence of anti-*Histoplasma* antibody in a general human population in URR, The Gambia, and explored potential risk factors associated with recent *Histoplasma* seropositivity.

## 2 Methods

### 2.1 Ethics statement

Ethical approval was granted by the Gambia Government/ Medical Research Council Joint Ethics Committee (SCC 26758) and the University of Liverpool Central University Research Ethics Committee (reference 8450).

### 2.2 Study design

**Fig. 1:**
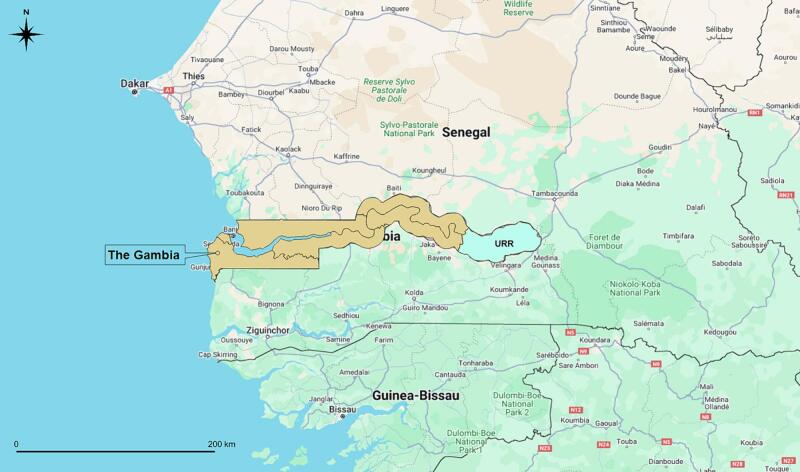
Local Government Areas (LGAs) of The Gambia, with Basse LGA highlighted, coterminous with Upper River Region (URR). Geodata were downloaded from Google Maps and provided by the Gambia Bureau of Statistics. Map was generated using QGIS 3.12.

This cross-sectional study, employing a random household sampling approach, was conducted from December 2022 to March 2023. The population of interest was a general population in Basse Local Government Area (LGA), coterminous with URR, The Gambia (Fig. 1). URR demonstrates a significant agricultural sector [24], with agricultural workers representing a potentially vulnerable population for *Histoplasma* exposure based on established occupational and environmental risk factors [25].

**Fig. 2:**
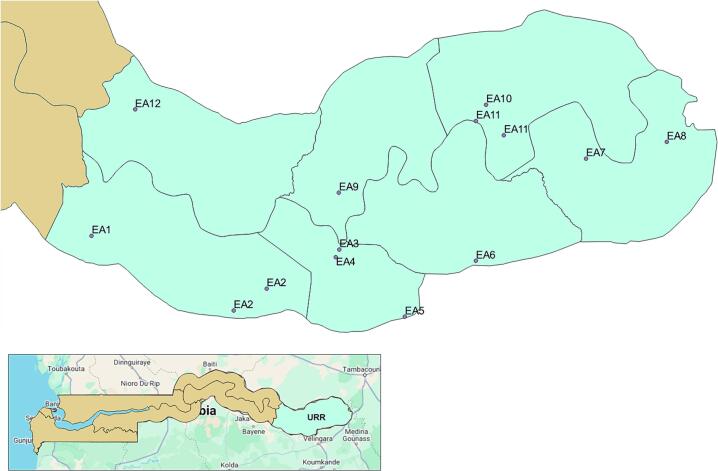
Settlement locations within selected Enumeration Areas (EA1–12) and district boundaries in Basse Local Government Area, coterminous with Upper River Region (URR). Geodata were downloaded from Google Maps and provided by the Gambia Bureau of Statistics. Maps were generated using QGIS 3.12.

Regional geographic divisions were characterised as: LGA, district, ward, settlement, Enumeration Area (EA), and household [26]. A household, or group of persons living together, reside within a compound. EAs represented study clusters, of which twelve were selected from a sampling frame of EAs in URR (*n* = 12/554, 2.2%; Fig. 2). A probability proportional to size approach was applied, using number of households per EA as the measure of size, and selection was stratified by urban and rural classification [24].

Sample size was calculated using a prevalence estimate of 15.5% [27] and a predicted between cluster variance of 5%. A confidence value of 95% and a desired absolute precision of 0.05 were applied. Per EA, target sample size was 25 individuals recruited from at least 3 households.

A random approach was designed to identify compounds, and households within selected compounds, tailored to individual EA layout (**S1 Fig.**).

### 2.3 Study participants

Any occupant of a randomly selected household aged ≥5 years was eligible to participate. In the event that the number of eligible household occupants was greater than required, participants were randomly selected by assigning numbers to all household occupants and blindly drawing assigned numbers from a bag until the target sample size was met.

Study information was written in English and disseminated verbally in the vernacular (Mandinka, Fula, Serahule or Wolof). Consenting individuals, or parents or legal guardians of assenting children (5–17 years), provided written signatory or witnessed thumbprint consent. Attending Village or Town Development Committee members, or Village Health Workers, who were literate, were identified to act as impartial witnesses of consent. Legal guardians consenting to participation of assenting children, completed a legal guardianship statement form. Study participants were assigned unique identification codes for pseudo-anonymisation and data collation purposes.

### 2.4 Data collection and blood sampling

A questionnaire designed for the study was delivered verbally to all participants by one appointed field assistant in the vernacular (**S2 Fig.**). Parents or legal guardians of assenting children were present to answer questions on behalf of the child or assist the child depending on individual comprehension. Questions were designed for this study, and related to the compound environment, animal contact and management, and occupation. Variables encompassed established or theoretically plausible risk factors for *Histoplasma* exposure based on current literature, and were contextually-appropriate. An extended questionnaire was delivered to the household representative, addressing household-level variables for which the response from all occupants within selected households was assumed the same.

Following the questionnaire, a clinical examination was performed by one appointed field nurse. A form was completed, addressing clinical history and examination findings (**S2 Fig.**). Variables included clinical symptoms and signs described in previous histoplasmosis case reports [[28], [29], [30]]. A single 5 mL venous blood sample was collected from each participant into plain and EDTA tubes. Sample tube barcodes were labelled with participant identification codes. As per study protocol, if indicated, recommendations and referrals to additional clinical services were made at the time of study interventions based on clinical examination findings.

Data were recorded offline on Research Electronic Data Capture (REDCap) application, using two digital devices, and uploaded daily to the study server.

### 2.5 Sample processing and serological testing

Blood collected in plain tubes was stored at room temperature for 30–60 min before placement in a cold box (with a cold life of 41 h at 8 °C at an ambient temperature of 43 °C), lined with coolant packs. Samples spun at 1000 x*g* for 15 min within four hours post-collection, were aliquoted into 2 mL cryovials labelled with participant identification codes and stored at −20 °C.

Serum samples were thawed and heat-treated (30 min at 56 °C). One IMMY Latex Agglutination *Histoplasma* test (LAT) was performed as per manufacturer guidelines per sample [31]. A graduated scale of reaction strengths was consulted to assign test results (from negative to four plus). Samples assigned a two plus or greater reaction strength were considered positive, indicative of an IgM response to *Histoplasma*, which was interpreted as presumptive evidence of recent *Histoplasma* exposure. Per ambiguous result, one repeat LAT test was performed.

### 2.6 Seroprevalence estimation

Apparent seroprevalence of anti-*Histoplasma* antibody was determined. True seroprevalence was estimated using published sensitivity and specificity values for a histoplasmin sensitised LAT of 62% and 97%, respectively, derived from cases encompassing acute primary *and* chronic pulmonary histoplasmosis [32]. Epitools interface and Clopper-Pearson (exact) test were employed to determine 95% Confidence Intervals (CIs) [33].

Seroprevalence at household and EA levels was determined by identifying households and EAs with at least one occupant demonstrating *Histoplasma* seropositivity, respectively.

### 2.7 Statistical analysis

Data were pseudo-anonymised and stored in password-protected .xlsx files. Study population characteristics were analysed using descriptive statistics, and compared to regional population statistics using 2022–23 population projection and 2013 Census data (acknowledgements to Jadama B, Gambia Bureau of Statistics) [34].

Univariable associations between *Histoplasma* seropositivity and categorical and continuous variables were examined using Odds Ratios (ORs) with 95% CIs, and associated *p*-values were calculated.

Phi and Spearman rank coefficients, and Pearson chi-squared test of association, were applied to explore correlations between variables. Correlation coefficients >0.7 with associated *p* < 0.05 were interpreted as evidence of correlations between variables. Subsequent *p*-value comparison on univariable analysis, and consideration of theoretical plausibility of correlated variables as risk factors for *Histoplasma* exposure, supported subsequent variable retention or exclusion.

Variables with a *p* < 0.20 on univariable analysis were selected for testing in a multivariable logistic regression model with seropositivity as the binary outcome. The model was built using a manual backwards-stepwise approach. Data points with a delta-beta of <−0.1 or > 0.1 were excluded, and the model output was re-examined to identify influential data points. Random effects were included by multi-level logistic regression analysis, using 2nd order predictive quasi-likelihood approximation method, to explore the effect of clustering of respondents at EA, ward and district levels. Regression coefficients, estimate *p*-values, and *z*-ratios were compared between single and multi-level models. Proportion of variance attributed to individual levels was calculated using the latent-variable approach. A residual plot at the EA level was constructed to examine the influence of individual EAs on model fit.

Statistical analyses and multi-level modelling were performed using IBM SPSS Statistics 27 and MLwiN 3.05 software, respectively.

## 3 Results

### 3.1 Descriptive data

Study participants (*n* = 298) represented 37 households across 12 EAs (2–4 households per EA), were 5 to 83 years (median = 27.0, IQR = 15.8–42.0), and comprised 165 females (55.4%). The median number of adults and children per household was 10 (range 2–25) and 12 (range 3–37), respectively. The age distribution was significantly different by gender (*p* = 0.01), with greater female representation in age categories 21–40 years (2.2 females: 1 male) and 41–60 years (1.6 females: 1 male). The majority of participants reported farming or domestic work as their primary occupation or household role. The odds of reporting domestic work were significantly higher in female participants (OR = 4.69 95% CI 2.82–7.78, *p* < 0.001), whereas there was no significant difference by sex on the odds of reporting farming as a primary role (**S1 Table**).

A median number of 10.0 adults (≥18 years) and 12.0 children (5–17 years) resided in selected households. At the household level, the majority of compound buildings comprised cement walls and floors, with iron roofs, and were surrounded by a concrete boundary wall which demonstrated an opening during the day. The majority of participants reported cooking with firewood, outside compound buildings, and sourced water from a borehole during dry and rainy seasons (**S2 Table**). Domestic animal or livestock contact, and observation of wildlife, were reported by the majority of participants (**S3 Table; S4 Table**). Household-level management practices of domestic animals or livestock in relation to the compound boundary, demonstrated day-to-night and seasonal variations (**S3 Table**). During the dry season, the majority of households kept domestic animals or livestock free-roaming during the day, but managed animals *inside* the compound at night. The majority of participants reported engagement in clearance, disposal or collection of animal manure (**S3 Table**).

Abnormal clinical signs, including chest pain, cough, dyspnoea and skin lesions, were reported by a minority of participants on clinical examination. A minority of participants were smokers or ex-smokers (**S5 Table**). Male and female participants demonstrated median Body Mass Index (BMI) of 18.5 and 21.5, respectively, which are comparable to BMI ranges from adult populations in West Africa [35].

Gender and age distributions of the study population were comparable to regional-level population statistics, with the exception of individuals aged 5–9 years which represented a greater proportion of the Basse LGA population ≥ 5 years (20.6%) than the study population (8.7%). The mean household size was greater amongst the study population compared to regional-level population statistics, in urban and rural EAs (**S6 Table**) [34].

### 3.2 Seroprevalence results

An apparent seroprevalence of 18.8% (*n* = 56/298, 95% CI 14.5–23.7%) was measured using the LAT, of which the majority of seropositive samples displayed a reaction strength of 2+ (*n* = 47/56, 83.9%; **S7 Table**). The estimated true seroprevalence was calculated as 26.8% (95% CI 19.8–34.9%) [33], adjusted for a published test sensitivity of 62% across cases encompassing acute *and* chronic pulmonary histoplasmosis. Using a test sensitivity of 100% measured in cases with *only* acute pulmonary histoplasmosis, this estimate decreases to 16.2% (95% CI 12.0–21.2%) which is comparable to the apparent seroprevalence [32]. Samples subject to a repeat LAT (*n* = 22/298, 7.38%) demonstrated 100% concordance between original and repeat test results.

At least one occupant per household demonstrated seropositivity in 67.6% of households (*n* = 25/37) and in 91.7% of EAs (*n* = 11/12). At the household level, the percentage of recruited participants demonstrating seropositivity ranged from 0.0% (*n* = 0/3 to 0/12 occupants) to 80.0% (*n* = 4/5 occupants).

### 3.3 Univariable analysis

On univariable logistic regression analysis, statistically significant associations (*p* < 0.05) were identified between LAT result and multiple demographic and environmental variables, including variables pertaining to animal contact and management (**S1-S3 Table**). Statistically significant differences in the odds of seropositivity were observed between EAs, wards and districts, which were explored as random effects on multi-level analysis (Table 1).

**Table 1:** Multi-level multivariable logistic regression model examining variable associations with *Histoplasma* seropositivity based on LAT results, amongst study participants (*n* = 298) in Upper River Region, The Gambia. Household, Enumeration Area, ward and district are included as random effects. Descriptive statistics, Odds Ratios (OR), 95% Confidence Intervals (CIs) and *p*-values, were calculated using MLwiN 3.05 software.

Variable	Frequency, *n* (%), total *N* = 298	*Histoplasma* seropositive, *n* (%), *N* = 56	*Histoplasma* seronegative, *n* (%), *N* = 242	Odds Ratio (95% Confidence Intervals)	*p-*value
Main effects					
Gender					
Male (ref)	133 (44.6)	20 (15.0)	113 (85.0)	1.00	
Female	165 (55.4)	36 (21.8)	129 (78.2)	2.41 (1.14–5.10)	0.021*
Age, years					
Median (IQR)	27.0 (15.8–42.0)	–	–	0.96 (0.93–0.98)	0.001*
Animal manure clearing/ disposal or collection					
No (ref)	75 (25.2)	5 (6.7)	70 (93.3)	1.00	
Yes	218 (73.2)	50 (22.9)	168 (77.1)	4.21 (1.38–12.90)	0.012*
No response	5 (1.7)	1 (20.0)	4 (80.0)	2.20 (0.17–28.45)	0.546
Animal management ^a^: Dry season, night					
Free compound entry/ exit (ref)	40 (13.4)	2 (5.0)	38 (95.0)	1.00	
Fenced/ tethered/ housed outside	65 (21.8)	11 (16.9)	54 (83.1)	4.70 (0.65–34.12)	0.126
Fenced/ tethered/ housed inside	193 (64.8)	43 (22.3)	150 (77.7)	10.72 (2.02–56.83)	0.005*
Random effects ^b^					
Household					
Variance (Standard Error)	0.00 (0.00)				
Enumeration Area					
Variance (Standard Error)	0.67 (0.42)				
Ward					
Variance (Standard Error)	0.00 (0.00)				
District					
Variance (Standard Error)	0.00 (0.00)				

* *p* < 0.05; ^a^ Domestic animal or livestock management in relation to compound boundary; ^b^ Random effects listed in order of smallest to largest geographic division.

No statistically significant associations were demonstrated between seropositivity and variables pertaining to either wildlife observation or management (**S4 Table**) or clinical status (**S5 Table**).

### 3.4 Multivariable logistic regression analysis

**Fig. 3:**
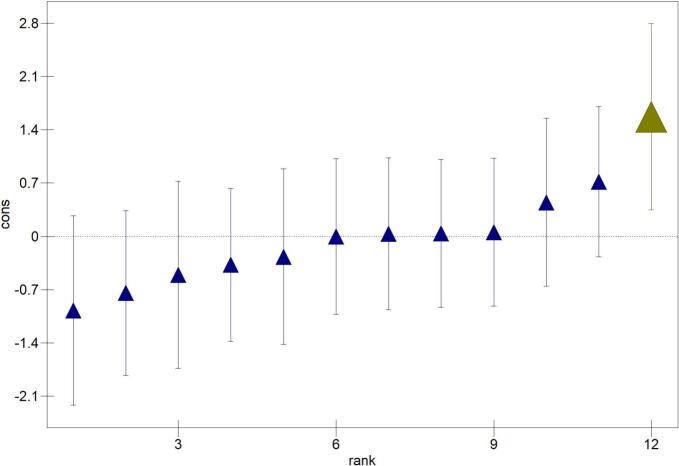
Plot of Enumeration Area (EA) level residuals (± 95% Confidence Intervals) of the 12 EAs, ordered by rank. The highest residual representing EA8 is highlighted in green. The plot was constructed using MLwiN 3.05 software. (For interpretation of the references to colour in this figure legend, the reader is referred to the web version of this article.)

A four-level multivariable model was proposed, which included random effects (in order of smallest to largest geographic division): household, EA, district, and ward (Table 1). Clustering by EA (variance = 0.67, SE 0.42) was demonstrated, indicating that 17.2% of variance in seropositivity is due to EA using the latent-variable approach. No evidence for clustering at the household, ward nor district levels was demonstrated (variance = 0.00, SE 0.00). Regression coefficients, *z*-ratios and *p-*values, of remaining variables in single- and multi-level models were comparable. A residual plot at the EA level demonstrated the variation and showed that EA8 was significantly different from the overall mean (Fig. 3). On univariable analysis, participants residing in this rural EA demonstrated significantly increased odds of *Histoplasma* seropositivity (compared to urban-classified EA3; **S1 Table**).

Odds of *Histoplasma* seropositivity were significantly greater amongst the following groups: female participants (OR = 2.41 95% CI 1.14–5.10, *p* = 0.02); participants involved in animal manure clearing, disposal or collection in the previous month (OR = 4.21 95% CI 1.38–12.90, *p* = 0.01); and participants managing domestic animals or livestock (fenced, tethered or housed) *inside* the compound at night during the dry season (OR = 10.72 95% CI 2.02–56.83, *p* = 0.01; Table 1). Increasing age was significantly associated with decreased odds of seropositivity (OR = 0.96 95% CI 0.93–0.98, *p* = 0.001). As study interventions were conducted during the dry season and a positive LAT result indicates recent *Histoplasma* exposure [36,37], only animal management variables pertaining to the dry season were retained in multivariable analyses.

On delta-beta analysis, no data points were determined to be influential on the model outcome.

## 4 Discussion

### 4.1 Main outcomes

A seroprevalence of anti-*Histoplasma* antibody of 18.8% (*n* = 56/298, 95% CI 14.5–23.7%) was demonstrated in a general population in URR, The Gambia. The model explaining the maximum variability in the data demonstrated statistically significant associations between seropositivity and the following demographic and environmental factors: gender; age; involvement in animal manure management; and domestic animal or livestock management approach in relation to the compound (at night during the dry season).

This study represents the first observational study in The Gambia, and more widely across West Africa, to examine the seroprevalence of anti-*Histoplasma* antibody in a general human population and explore risk factors for *Histoplasma* exposure in a community setting.

In The Gambia, historic case reports of histoplasmosis describe a four-year old child presenting with cutaneous lesions and a lesion of the femur [16], and an adult with adenopathy and oral lesions, with a recent travel history to The Gambia [38]. The patients' medical histories, immunocompetence, or environmental exposures, are not described. Frequent *Histoplasma* exposure amongst active TB cases in The Gambia was demonstrated using a LAT, with a measured seropositivity of 28.8% (*n* = 15/52, 95% CI 17.1–43.1%) [39]. Thus, robust *Histoplasma*-focussed research applying a One Health approach was warranted to estimate the true *Histoplasma* burden in The Gambia, and to explore risk factors for exposure at this human-animal-environment interface. The study area represented by this study is restricted to URR. Additional studies are warranted in other regions to determine national seroprevalence, and to explore inter-regional sociodemographic and geoclimatic variations which may affect odds of *Histoplasma* exposure.

The apparent seroprevalence measured in this study is comparable to a previously measured seroprevalence of 15.5% (*n* = 104/670, 95% CI 12.9–18.5%) in a general population in rural western Kenya, an area represented by smallholder, mixed crop-livestock production [27]. In URR, The Gambia, the agricultural sector is characterised by mixed subsistence farming systems, primarily crop production [40]; thus, inferences about shared occupational or environmental exposures between these geographic regions are justified.

Occupational and recreational activities involving soil disruption are described as risk factors for *Histoplasma* exposure [25]. In URR, 67.3% and 85.1% of economically active males and females, respectively, have been identified as agricultural workers [24]; thus, this population may be at increased risk of exposure. Furthermore, The Gambia's working equid population has demonstrated high anti-*Histoplasma* antibody seropositivity (Cornell T R et al., unpublished) and nationwide, the largest number of horses (37.6%) and donkeys (36.8%) reside in URR [41]. Thus, current evidence indicates that *Histoplasma* exists at this human-animal-environment interface.

For every 1-year increase in age, the odds of *Histoplasma* seropositivity decreased by 4.0%. This statistically significant association concurs with previous outcomes in general and patient populations [27,39]. During an initial antigen challenge, IgM represents the first immunoglobulin isotype expressed by immature B cells in the immunocompetent host, and is the target of the LAT. On sustained or recurrent antigen exposure, isotype switching results in an immune response characterised by IgG and IgA production [36,42]. Based on evidence suggesting *Histoplasma* is endemic in The Gambia, initial exposure in immunocompetent children may result in an IgM response detectable by LAT, which diminishes with age with chronic exposure. Investigation of age-related immunocompetence and exposures is warranted. Correlations were demonstrated between age and participation in both animal manure management (*p* = 0.02), and feeding or grazing of animals (*p* = 0.01). These outcomes concurred with anecdotal evidence from participants, describing the contribution of children to animal management activities (Study participants, personal communication).

Female participants demonstrated significantly increased odds of *Histoplasma* exposure; thus, investigation of gender-related exposure to potential risk factors is warranted. A significant correlation was measured between participant sex and reporting domestic work as a primary role (*p* < 0.001). Increased time in the compound could result in more frequent exposure of female participants to environmental reservoirs of *Histoplasma*, including animal manure managed within the compound boundary. Age distributions by sex were significantly different; thus, investigation of age-related exposures to *Histoplasma* and immunocompetence is warranted.

Odds of seropositivity were significantly higher in participants involved in animal manure management, supported by evidence of reservoirs of *Histoplasma* in soil and avian faeces [[43], [44], [45]]. Odds of seropositivity were significantly higher in participants from households managing animals inside the compound boundary at night, compared to compounds where animals could freely enter or exit. This might reflect increased frequency of direct human-animal contact within the compound and associated animal management activities, including manure collection which we hypothesise to be a *Histoplasma* reservoir.

Longitudinal research is warranted to examine the impact of seasonal variations in animal contact and management strategies, and engagement in farming activities, on *Histoplasma* exposure. During the day, the majority of households reported that domestic animals or livestock were kept free range in the dry season (*n* = 17/37, 45.9%), but housed, tethered or fenced outside the compound in the rainy season (*n* = 22/37, 59.5%). Thus, we speculate that *Histoplasma* exposure may be greater during the rainy season.

Variables pertaining to domestic animal contact and donkey ownership lost significance on inclusion of household, EA, ward and district as random effects. Furthermore, equid contact was greater in rural compared to urban EAs (*p* < 0.001); and median number of donkeys per household was greater in rural versus urban EAs (*p* < 0.001). Geographic variations in seroprevalence demonstrated on univariable analysis and by residual plots at the EA level warrant investigation, to explore predictors of *Histoplasma* exposure in rural versus urban EAs. These study outcomes allude to a complex interplay between animal and environmental factors in rural The Gambia, and support further investigation of both the individual and cumulative impact of inter-related variables on the odds of *Histoplasma* exposure. In line with evolving definitions of One Health, the significance of *Histoplasma* reservoirs within a larger social-ecological system warrants investigation. Characterisation of isolates of human, animal and environmental origin is warranted to investigate *Histoplasma* transmission dynamics in a community setting and to explore the zoonotic potential of this high priority fungal pathogen.

### 4.2 Limitations

Consideration of relationships between occupants was crucial to avoid disrupting established hierarchies at compound and household levels. These dynamics presented challenges for implementing a random sampling approach, as household heads may themselves elect occupants to participate prior to collection of informed consent. Despite potential deviations from a random approach, comparison with regional population statistics demonstrated that the study population was comparable with respect to age and sex distributions.

17.2% of variance in seropositivity was attributable to EA, which is higher than the predicted between cluster variance of 5%. Underestimation of the degree of clustering reduces power. The reported between cluster variance provides a baseline for future sample size approximations, although this may vary depending on context and sampling strategy.

A positive LAT result provides presumptive evidence of an IgM response to *Histoplasma*, mounted 2–6 weeks post-exposure. Study participants tested prior to 2 weeks post-exposure or after the IgM response has diminished may present false negative results [36,46], including potential cases of latent or resolved nodular pulmonary histoplasmosis [47]. This is reflected in variable sensitivity values for a histoplasmin sensitised LAT of 100% and 45.7% in cases of acute primary and chronic pulmonary histoplasmosis, respectively [32]. Evidence of false negative serological test results in immunocompromised patients has been documented, as a result of the hosts' inability to mount a normal immune response to *Histoplasma* [48,49]; thus, the seroprevalence estimate may be an underestimate in the minority of study participants reporting clinical abnormalities. HIV infection status was not determined in this study; however, national 2022 estimates report an HIV prevalence of 1.4% in adults aged 15–49 years [50], equating to 2–3 study participants within this age range (15–49 years: *n* = 176/298).

The LAT was selected as a practical qualitative serological test which required minimal benchtop equipment to provide evidence of recent *Histoplasma* exposure. Conducting a comparative immunodiagnostic or antigen test is warranted to confirm results, and to differentiate recent and historic antibody responses to *Histoplasma*.

In The Gambia, chronic aflatoxin exposure as a result of groundnut and maize crop contamination with *Aspergillus* spp. has been documented [51]. Exposures to other endemic mycoses may result in LAT cross-reactions, warranting pan-fungal diagnostic tests and targeted environmental sampling to identify prevalent fungal species [52].

## 5 Conclusions

Baseline evidence is provided for frequent recent *Histoplasma* exposure in URR, The Gambia, and plausible demographic and environmental risk factors for seropositivity. The study addresses an evidence gap on the burden of *Histoplasma* exposure in one region of The Gambia; and investigates the need for active surveillance in communities exposed to potential *Histoplasma* reservoirs. Future studies could utilise this evidence to design risk prediction models for *Histoplasma* exposure, and develop context-specific risk mitigation approaches, such as education around animal management in the compound.

To disentangle *Histoplasma* transmission dynamics in community settings in The Gambia, the following research directions are warranted: (i) identification of *Histoplasma* reservoirs; (ii) comparative phylogenetic analysis of *Histoplasma* isolates from human, animal and environmental origins; and (iii) ascertainment of public health implications of reservoirs for general and immunocompromised human populations, and the zoonotic potential of characterised *Histoplasma* spp.

The following are the supplementary data related to this article.

**Supplementary Fig. S1:**
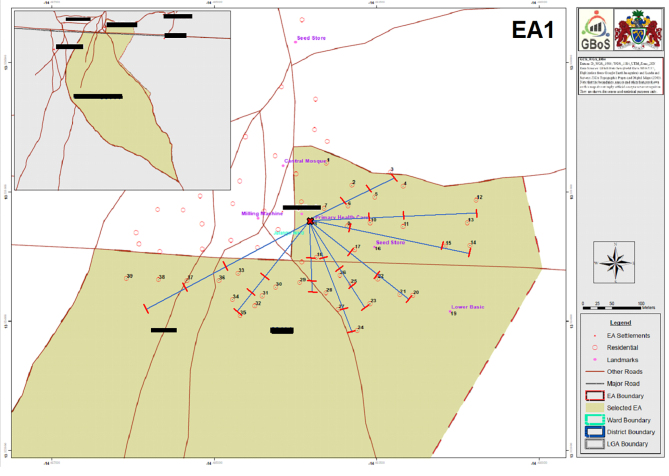
Example of annotated Enumeration Area (EA) topography map used to identify EA boundaries, landmarks and compounds, and approximate distances from the centre to the periphery of the EA. Maps were provided by the Gambia Bureau of Statistics (GBoS) from the 2013 Population and Housing Census.

Supplementary Fig. S2 Questionnaire and Clinical history and examination form, on Research Electronic Data Capture (REDCap) application. Supplementary Fig. S2

Supplementary Table S1 Univariable logistic regression analysis results, examining associations between *Histoplasma* seropositivity based on LAT result and demographic variables, amongst study participants (*n* = 298) in Upper River Region, The Gambia. Frequencies (*n*), percentages (%), Odds Ratios (OR), 95% Confidence Intervals (CIs) and *p*-values, were calculated using IBM SPSS Statistics 27. Supplementary Table S1

Supplementary Table S2 Univariable logistic regression analysis results, examining associations between *Histoplasma* seropositivity based on LAT result and environmental variables, amongst study participants (*n* = 298) in Upper River Region, The Gambia. Frequencies (*n*), percentages (%), Odds Ratios (OR), 95% Confidence Intervals (CIs) and *p*-values, were calculated using IBM SPSS Statistics 27. Supplementary Table S2

Supplementary Table S3 Univariable logistic regression analysis results, examining associations between *Histoplasma* seropositivity based on LAT result and animal contact and management variables, amongst study participants (*n* = 298) in Upper River Region, The Gambia. Frequencies (*n*), percentages (%), Odds Ratios (OR), 95% Confidence Intervals (CIs) and *p*-values, were calculated using IBM SPSS Statistics 27. Supplementary Table S3

Supplementary Table S4 Univariable logistic regression analysis results, examining associations between *Histoplasma* seropositivity based on LAT result and wildlife observation variables, amongst study participants (*n* = 298) in Upper River Region, The Gambia. Frequencies (*n*), percentages (%), Odds Ratios (OR), 95% Confidence Intervals (CIs) and *p*-values, were calculated using IBM SPSS Statistics 27. Supplementary Table S4

Supplementary Table S5 Univariable logistic regression analysis results, examining associations between *Histoplasma* seropositivity based on LAT result and clinical variables, amongst study participants (*n* = 298) in Upper River Region, The Gambia. Frequencies (*n*), percentages (%), Odds Ratios (OR), 95% Confidence Intervals (CIs) and *p*-values, were calculated using IBM SPSS Statistics 27. Supplementary Table S5

Supplementary Table S6 Baseline demographic characteristics of study sample and Basse LGA population, using the Gambia Bureau of Statistics (GBoS) 2022–3 population projection data and 2013 Population and Housing Census data. Supplementary Table S6

Supplementary Table S7 The frequency distribution of IMMY Latex Agglutination-*Histoplasma* test results for study participants (*n* = 298), categorised by reaction strength, and result interpretation. Supplementary Table S7

Supplementary Table S8 Univariable logistic regression analysis results, examining associations between *Histoplasma* seropositivity based on LAT result and clinical variables, amongst study participants (*n* = 298) in Upper River Region, The Gambia. Frequencies (*n*), percentages (%), Odds Ratios (OR), 95% Confidence Intervals (CIs) and *p*-values, were calculated using IBM SPSS Statistics 27. Supplementary Table S8

Supplementary Table S9 Baseline demographic characteristics of study sample and Basse LGA population, using the Gambia Bureau of Statistics (GBOS) 2022–3 population projection data and 2013 Population and Housing Census data. Supplementary Table S9

Supplementary Table S10 The frequency distribution of IMMY Latex Agglutination-*Histoplasma* test results for study participants (*n* = 298), categorised by reaction strength, and result interpretation. Supplementary Table S10

## Funding

The study was supported by 10.13039/100010269Wellcome Trust (206638/Z/17/Z to CES) and a 10.13039/501100000836University of Liverpool-funded PhD studentship (to TRC).

## CRediT authorship contribution statement

**Tessa Rose Cornell:** Conceptualization, Data curation, Formal analysis, Investigation, Methodology, Project administration, Validation, Visualization, Writing – original draft, Writing – review & editing. **Bakary Conteh:** Investigation, Project administration, Resources, Supervision, Writing – review & editing. **Lamin Drammeh:** Investigation. **Foday Jeffang:** Investigation. **Alkali Kijera:** Investigation. **Mustapha Jarju:** Investigation. **Mehrab Karim:** Data curation. **Pa Ousman Ceesay:** Methodology. **Ebrima Manneh:** Investigation. **Regional Health Directorate Upper River Region:** Investigation **Dan G. Wootton:** Supervision, Writing – review & editing, Methodology. **Gina Pinchbeck:** Supervision, Writing – review & editing, Methodology. **Claire Elizabeth Scantlebury:** Conceptualization, Funding acquisition, Methodology, Project administration, Supervision, Validation, Writing – review & editing.

## Declaration of competing interest

None.

## Data Availability

Anonymised data available upon request; will submit to University of Liverpool data repository
