# Supplementary material for: Histoplasma seropositivity and environmental risk factors for exposure in a general population in Upper River Region, The Gambia: A cross-sectional study
Source: One Health. 2024 Mar 27;18:100717. doi: 10.1016/j.onehlt.2024.100717 (PMC10992707; doi:10.1016/j.onehlt.2024.100717)
Supplement: Supplementary Fig. S2 — Questionnaire and Clinical history and examination form, on Research Electronic Data Capture (REDCap) application. [file mmc2.pdf]

Consent and Contact details

Study Participant ID: __________________________________

A. Contact details

1. Has the individual given voluntary informed consent to study participation?
   - ◯ Yes
   - ◯ No

2. Image of signed informed consent document

3. Household ID __________________________________

4. EA code
   - ◯ 80114
   - ◯ 80201
   - ◯ 81119
   - ◯ 81147
   - ◯ 81212
   - ◯ 82135
   - ◯ 83106
   - ◯ 83219
   - ◯ 84117
   - ◯ 85111
   - ◯ 85127
   - ◯ 86124

5. Settlement
   - ◯ Nawdeh
   - ◯ Taibatu
   - ◯ Baja Kunda
   - ◯ Boro Modi Banni
   - ◯ Fata Tenda
   - ◯ Heramakono & Chemenbugu
   - ◯ Garawol
   - ◯ Dingiri
   - ◯ Sare Pisaru
   - ◯ Manneh Kunda
   - ◯ Basse Santo-Su
   - ◯ Sandy Kunda
   - ◯ Sare Alhagie Sorry (Jallow Kunda)
   - ◯ Sare Bondo (Gambissara Fula)

6. Ward
   - ◯ Julangel
   - ◯ Gambissara
   - ◯ Basse
   - ◯ Sabi
   - ◯ Dampha Kunda
   - ◯ Garawol
   - ◯ Koina
   - ◯ Sutokonding
   - ◯ Baja Kunda
   - ◯ Diabugu

7. District	◯ Basse<br>◯ Wuli East<br>◯ Jimara<br>◯ Tumana<br>◯ Kantora<br>◯ Wuli West<br>◯ Sandu
8. First name	________________________
9. Surname or family name	________________________
10. Contact number<br>If participant doesn't have contact number, give contact number for household representative	________________________
11. Preferred contact method(s) (select all options that apply)	☐ SMS text<br>☐ WhatsApp message<br>☐ Landline call<br>☐ WhatsApp call<br>☐ Other
11a. Specify other	________________________

B. FORM COMPLETION

12. Date	________________________
13. Time	________________________
14. ID of attending research team member who completed this form	________________________

Questionnaire Form

1. Attending research team member ID

____

2. Language

◯ Mandinka
◯ Wolof
◯ Fula/Pula
◯ Serahule
◯ English
◯ Other

2a Specify other

____

3. Date:

____

4. Start time:

____

Prior to starting the questionnaire, please read the following statement to the study participant:

Thank you for consenting to take part in this study. This is the first part of your contribution to the study. I will ask you a series of questions about the environments in which you live and work. Please answer all to the best of your knowledge, and you are free to not answer any questions you may not be comfortable with. Please let me know if there is anything you do not understand, or would like me to repeat, any of the following questions.

Are you happy for me to begin?

◯ Yes
◯ No

If the study participant answers No, answer any further questions the participant has and/or if the participant chooses to withdraw, please complete a withdrawal form.

5. Are you the household head or representative?

◯ Yes
◯ No

A. COMPOUND ENVIRONMENT

This part of the questionnaire is to be answered by the compound occupant identified as the household head or representative

6. How many adults (18 years and older) are in the household?

____

7. How many children (less than 18 years) are in the household?

____

8. In the building(s) you live in, what materials are the floors constructed of?

☐ Earth
☐ Compacted mud
☐ Cement
☐ Tiles
☐ Other (please specify below)

8a. Specify other

____

9. In the building(s) you live in, what materials are the walls constructed of?

- ☐ Mud
- ☐ Unburnt bricks
- ☐ Burnt bricks
- ☐ Cement
- ☐ Other (please specify below)

9a. Specify Other

____

10. In the building(s) you live in, what materials are the roofs constructed of?

- ☐ Iron/ corrugated iron
- ☐ Thatch/ grass
- ☐ Wood
- ☐ Cement
- ☐ Cardboard
- ☐ Other (please specify below)

10a. Specify Other

____

11. Is there a fence or boundary identifying the area of your compound where you live?

- ◯ Yes
- ◯ No

12. What material is the fence or boundary constructed of?

- ☐ Iron/ corrugated iron
- ☐ Wooden posts
- ☐ Plants/ shrubs
- ☐ Concrete
- ☐ Other (please specify below)

12a. Specify other

____

13. Is there an opening in the fence or boundary of your compound where you live?

- ◯ Yes
- ◯ No

14. Is there an opening in the fence or boundary during the day or at night?

- ◯ During day
- ◯ During night
- ◯ Both

15. What prevents domestic animals, livestock, or wild animals from entering or exiting the compound freely?

- ☐ Animals can enter or exit household freely
- ☐ Fence or boundary is closed by gate/fence/ door
- ☐ Domestic animals or livestock are tethered
- ☐ Domestic animals or livestock are housed
- ☐ Domestic animals or livestock are fenced
- ☐ Other (please specify below)

15a. Specify other

____

16. In the building(s) you live in, do you have a method to ventilate (keep air circulating in) the household?

- ☐ No ventilation system
- ☐ Direct opening in wall or roof (including windows)
- ☐ Air conditioning
- ☐ Other (please specify below)

16a. Specify other

____

17. Where is the primary location for cooking food?	◯ Inside building(s) you live in<br>◯ Outside building(s) you live in
18. What is the primary fuel you use for cooking food?	◯ Electricity<br>◯ Firewood<br>◯ LPG (liquefied petroleum gas)<br>◯ Charcoal<br>◯ Sawdust<br>◯ Other (please specify below)
18a. Specify other	__________________________
19. In the compound, what is your primary source of water for drinking in the DRY season?	☐ Borehole located inside compound area<br>☐ Borehole located outside compound area<br>☐ Covered well located inside compound area<br>☐ Covered well located outside compound area<br>☐ Uncovered well located inside compound area<br>☐ Uncovered well located outside compound area<br>☐ Pond or dam<br>☐ River<br>☐ Spring or stream<br>☐ Piped into compound<br>☐ Roof capture<br>☐ Other (please specify below)
19a. Specify other	__________________________
20. In the compound, what is your primary source of water for drinking in the RAINY season?	☐ Borehole located inside compound area<br>☐ Borehole located outside compound area<br>☐ Covered well located inside compound area<br>☐ Covered well located outside compound area<br>☐ Uncovered well located inside compound area<br>☐ Uncovered well located outside compound area<br>☐ Pond or dam<br>☐ River<br>☐ Spring or stream<br>☐ Piped into compound<br>☐ Roof capture<br>☐ Other (please specify below)
20a. Specify other	__________________________
21. What time does it take you or a member of your household to walk to your primary source of water? (Minutes walking)	________________________________
22. In the compound, do you treat your water before drinking it?	◯ Yes<br>◯ No
23. What primary method of water treatment do you use?	☐ Boiling<br>☐ Add chlorine<br>☐ Add iodine<br>☐ Water disinfectant/bleach<br>☐ Filter<br>☐ Other (please specify below)

23a. Specify other

____

24. Do you observe flies in the building(s) you live in?

◯ Yes
◯ No

B. ANIMAL EXPOSURE

Contact with domestic animals or livestock may occur in the environments where you live or work. We are interested to hear about which types of animals you may be in contact with.

25. Do you have contact with any domestic animals or livestock? These include, dogs, cats, poultry, horses, donkeys, cattle, sheep, goats, and pigs, within your compound?

◯ Yes
◯ No

26. Which domestic animals or livestock are you in contact with in your compound?

☐ Poultry
☐ Cattle
☐ Horses/ donkeys/mules
☐ Sheep/ goats
☐ Pigs
☐ Dogs
☐ Cats
☐ Other (please specify below)
☐ Other 2 (please specify below)

26a. Specify other

____

26b. Specify other 2

____

27. For each species named above, please indicate how often are you in contact with domestic animals or livestock in your compound?

	Daily	Weekly	Monthly	Less often than monthly	Previously but not anymore*
Poultry	◯	◯	◯	◯	◯
Cattle	◯	◯	◯	◯	◯
Horses	◯	◯	◯	◯	◯
Donkeys	◯	◯	◯	◯	◯
Mules	◯	◯	◯	◯	◯
Sheep/ goats	◯	◯	◯	◯	◯
Pigs	◯	◯	◯	◯	◯
Dogs	◯	◯	◯	◯	◯
Cats	◯	◯	◯	◯	◯
[qs_ani_contract_othr]	◯	◯	◯	◯	◯
[qs_ani_contract_othr_2]	◯	◯	◯	◯	◯

* Previously but not anymore can be specified as more than 1 month previously, but not anymore

28a. How many horses do you or members of your household currently own?	______________________
28b. How many Donkeys do you or members of your household currently own?	______________________
28c. How many Mules do you or members of your household currently own?	______________________
29. What is the main use of your horses?	__________________________
30. What is the main use of your donkeys?	__________________________
31. What is the main use of your mules?	__________________________
32. Do domestic animals or livestock have access to the building(s) you sleep in?	◯ Yes<br>◯ No
33. In the RAINY season, during the DAY, where are domestic animals or livestock animals kept in relation to the compound you live in?	☐ Animals can enter or exit compound freely<br>☐ Fenced area outside compound<br>☐ Fenced area inside compound<br>☐ Tethered outside compound<br>☐ Tethered inside compound<br>☐ Housed area outside compound*<br>☐ Housed area inside compound*<br>☐ Other (please specify below)
* Housed area may include permanent or temporary structures (e.g. tent	
33a. Animals can enter or exit compound freely (Specify which animals separated by a comma)	______________________
33b. Fenced area outside compound (Specify which animals separated by a comma)	______________________
33c. Fenced area inside compound (Specify which animals separated by a comma)	______________________
33d. Tethered outside compound (Specify which animals separated by a comma)	______________________
33e. Tethered inside compound (Specify which animals separated by a comma)	______________________
33f. Housed area outside compound (Specify which animals separated by a comma)	______________________
33g. Housed area inside compound (Specify which animals separated by a comma)	______________________

33h. Specify other (place where animals are kept) ______________________________

33i. [qs_ani_entr_othr_plac] (Specify which animals separated by a comma) ______________________________

34. In the RAINY season, during the NIGHT, where are domestic animals or livestock animals kept in relation to the compound you live in?

- ☐ Animals can enter or exit compound freely
- ☐ Fenced area outside compound
- ☐ Fenced area inside compound
- ☐ Tethered outside compound
- ☐ Tethered inside compound
- ☐ Housed area outside compound*
- ☐ Housed area inside compound*
- ☐ Other (please specify below)

* Housed area may include permanent or temporary structures (e.g. tent

34a. Animals can enter or exit compound freely (Specify which animals separated by a comma) ______________________________

34b. Fenced area outside compound (Specify which animals separated by a comma) ______________________________

34c. Fenced area inside compound (Specify which animals separated by a comma) ______________________________

34d. Tethered outside compound (Specify which animals separated by a comma) ______________________________

34e. Tethered inside compound (Specify which animals separated by a comma) ______________________________

34f. Housed area outside compound (Specify which animals separated by a comma) ______________________________

34g. Housed area inside compound (Specify which animals separated by a comma) ______________________________

34h. Specify other (place where animals are kept) ______________________________

34i. [qs_ani_entr_othr_ng] (Specify which animals separated by a comma) ______________________________

35. In the DRY season, during the DAY, where are domestic animals or livestock animals kept in relation to the compound you live in?

- ☐ Animals can enter or exit compound freely
- ☐ Fenced area outside compound
- ☐ Fenced area inside compound
- ☐ Tethered outside compound
- ☐ Tethered inside compound
- ☐ Housed area outside compound*
- ☐ Housed area inside compound*
- ☐ Other (please specify below)

* Housed area may include permanent or temporary structures (e.g. tent

35a. Animals can enter or exit compound freely (Specify which animals separated by a comma) ______________________________

35b. Fenced area outside compound (Specify which animals separated by a comma)	______________________
35d. Tethered outside compound (Specify which animals separated by a comma)	______________________
35c. Fenced area inside compound (Specify which animals separated by a comma)	______________________
35e. Tethered inside compound (Specify which animals separated by a comma)	______________________
35f. Housed area outside compound (Specify which animals separated by a comma)	______________________
35g. Housed area inside compound (Specify which animals separated by a comma)	______________________
35h. Specify other (place where animals are kept)	______________________
35i. [qs_ani_entr_othr_dry_day] (Specify which animals separated by a comma)	______________________
36. In the DRY season, during the NIGHT, where are domestic animals or livestock animals kept in relation to the compound you live in?	☐ Animals can enter or exit compound freely<br>☐ Fenced area outside compound<br>☐ Fenced area inside compound<br>☐ Tethered outside compound<br>☐ Tethered inside compound<br>☐ Housed area outside compound*<br>☐ Housed area inside compound*<br>☐ Other (please specify below)
* Housed area may include permanent or temporary structures (e.g. tent	
36a. Animals can enter or exit compound freely (Specify which animals separated by a comma)	______________________
36b. Fenced area outside compound (Specify which animals separated by a comma)	______________________
36c. Fenced area inside compound (Specify which animals separated by a comma)	______________________
36d. Tethered outside compound (Specify which animals separated by a comma)	______________________
36e. Tethered inside compound (Specify which animals separated by a comma)	______________________
36f. Housed area outside compound (Specify which animals separated by a comma)	______________________
36g. Housed area inside compound (Specify which animals separated by a comma)	______________________

36h. Specify other (place where animals are kept)	__________________________________
36i. [qs_ani_entr_othr_dry_ng] (Specify which animals separated by a comma)	__________________________________
37. What materials are the floors of the housed areas constructed of?	☐ Earth<br>☐ Compacted mud<br>☐ Cement<br>☐ Other (please specify below)
37a. Specify other	__________________________________
38. What materials are the walls of the housed areas constructed of?	☐ Mud<br>☐ Unburnt bricks<br>☐ Burnt bricks<br>☐ Cement<br>☐ Other (please specify below)
38a. Specify Other	__________________________________
39. What materials are the roof of the housed areas constructed of?	☐ Iron/ corrugated iron<br>☐ Thatch/ grass<br>☐ Wood<br>☐ Cement<br>☐ Cardboard<br>☐ Other (please specify below)
39a. Specify Other	__________________________________
40. Are the housed areas fixed or temporary structures?	○ Fixed (for example, shed or stable)<br>○ Temporary (for example, tent)<br>○ Both
41. Do you collect droppings/ manure from domestic animals or livestock from the compound environment?	○ Yes<br>○ No
42. Are droppings/ manure from domestic animals or livestock stored in the compound environment?	○ Yes<br>○ No
43. Which domestic animals or livestock do you collect droppings/ manure from within the compound?	☐ Poultry<br>☐ Cattle<br>☐ Horses/ donkeys/mules<br>☐ Sheep/ goats<br>☐ Pigs<br>☐ Dogs<br>☐ Cats<br>☐ Other (please specify below)
43a. Specify other	__________________________________

Observation of wild animals may occur in the environments where you live or work.

44. In the last seven days, have you observed wild animals within your compound?

◯ Yes
◯ No

45. In the last seven days, which wild animals have you observed within your compound?

☐ Bats
☐ Wild birds
☐ Rats
☐ Monkeys
☐ Lizards
☐ Other (please specify below)
☐ Other 2 (please specify below)

45a. Specify other

____

45b. Specify other

____

46. For each wild animal observed, please indicate how often you observe them in your compound environment?

	Daily	Weekly	Monthly	Less often than monthly	Previously but not anymore*
Bat	◯	◯	◯	◯	◯
Wild birds	◯	◯	◯	◯	◯
Rats	◯	◯	◯	◯	◯
Monkeys	◯	◯	◯	◯	◯
Lizards	◯	◯	◯	◯	◯
[qs_wild_ani_obsrv_othr]	◯	◯	◯	◯	◯
[qs_wild_ani_obsrv_othr_2]	◯	◯	◯	◯	◯

* Previously but not anymore can be specified as more than 1 month previously, but not anymore

47. Do you observe droppings/ faeces from wild animals in your compound environment?

◯ Yes
◯ No

48. Which wild animals are producing the droppings/ faeces in your compound environment?

☐ Bats
☐ Wild birds
☐ Rats
☐ Monkeys
☐ Lizards
☐ Hyenas
☐ Other (please specify below)
☐ Unknown

48a. Specify other

____

49. What is your view of wildlife within the compound?

____

Additional prompt question may be used: Does wildlife within the compound concern
you?

50. Do you implement any practices/ measures to reduce wildlife within the compound?

____

51. In the previous one month have you carried out any of the following activities?

	Yes	No
Hunting of wild animals	○	○
Slaughter of animals?	○	○
Skinning or butchering of animals?	○	○
Clearing away/ disposing of animal manure?	○	○
Collecting animal manure?	○	○
Burial of dead animals?	○	○
Feeding or grazing animals?	○	○

52. If you have carried out any of the activities in question 46, which animal species or type were involved for each activity carried out?

Hunting of wild animals?

____

Slaughter of animals?

____

Skinning or butchering of animals?

____

Clearing away/ disposing of animal manure?

____

Collecting animal manure?

____

Burial of dead animals?

____

Feeding or grazing animals?

____

C. DEMOGRAPHIC CHARACTERISTICS

The word occupation means your principal work. This may include the main way in which you earn money or the main role you have in the household

53. What is your primary occupation or role in the household? __________________________________

54. Does your primary occupation involve building or construction work activities?
- ◯ Yes
- ◯ No

55. Does your main occupation involve soil excavation activities? This includes farming.
- ◯ Yes
- ◯ No

56. What ethnic/ tribal group are you?
- ☐ Mandinka
- ☐ Jahanka
- ☐ Serahule
- ☐ Wolof
- ☐ Jola
- ☐ Fula/ Pula
- ☐ Serer
- ☐ Manjago
- ☐ Other

56a. Specify Other ____________________________

When you have finished the questionnaire, including answering any questions the study participant may have, please read the following statement to the study participant:
“This is the end of the household questionnaire. We will now ask some questions about your health and perform a short clinical exam.”

D. FORM COMPLETION

57. End time ____________________________

58. ID of attending research team member who completed this form ____________________________

Clinical History And Examination Form

1. ID of Attending research team member

____

2. Language

◯ Mandinka
◯ Wolof
◯ Fula/Pula
◯ Serahule
◯ English
◯ Other

2a. Specify other

____

3. Date:

____

4. Start time:

____

The clinical examination will be conducted in the compound. Identify a private space within the compound building or set up the privacy screen within the compound boundary. Please read the following statement to the study participant:

I will now ask you some questions and perform a short clinical examination. Please answer all of my questions to the best of your knowledge. I strongly encourage you to tell me if you do not understand, or would like me to repeat, any of the following questions.

5. Are you happy for me to begin?

◯ Yes
◯ No

If the study participant answers No, answer any further questions the participant has and/or if the participant chooses to withdraw, please complete a withdrawal form.

A. PERSONAL DETAILS

6. Sex

◯ Male
◯ Female

7. Age (In Years)

____

B. CLINICAL HISTORY

'Ask the following clinical history questions and mark the study participant answers

8. Do you currently have any of the following clinical symptoms?

	Yes	No
Cough	◯	◯
Shortness of breath	◯	◯
Chest pain	◯	◯
Fever	◯	◯
Night sweats	◯	◯
Loss of appetite	◯	◯
Weight loss	◯	◯
Skin lesions	◯	◯
Lesions in the mouth	◯	◯
Muscle pain	◯	◯
Joint pain	◯	◯

9. What is the nature of the cough?

☐ With mucus
☐ Without mucus (dry)
☐ Wheeze

10. Do you cough up blood?

◯ Yes
◯ No

11. Of the following options regarding smoking, what is your current status?

◯ Active smoker
◯ Ex-smoker
◯ Never smoked

11a. What duration of time did you smoke for?

____

11b. On average how many cigarettes or any other types of tobacco products are you smoking or did you smoke, per day?[Free text]

____

11c. What type of tobacco products do or did you smoke? Examples may include cigarettes, cigars or pipes? [Free text]

____

12. Before your involvement in the study, had you heard of the disease histoplasmosis?

(Hints: Describe non-specific respiratory signs, caused by fungus, may be present in environmental reservoirs e.g. soil, bird and bat faeces, same fungus causes Epizootic Lymphangitis [use local names] in horses and donkey)

◯ Yes
◯ No

12a. Where did you hear about this disease?

____

C. GENERAL PARAMETERS

13. Temperature (in °C) __________________________________

14. Respiratory rate (breaths per minute) __________________________________

15. Weight (kilograms) __________________________________

16. Height (centimeters): __________________________________

D. CLINICAL EXAMINATION

Please determine the following on general examination and mark the answers that apply

17. Observe the study participant. Are they displaying signs of any of the following?

	Yes	No
Chest pain?	◯	◯
Dyspnoea (breathing problems)?	◯	◯
Cough?	◯	◯
Abnormal chest auscultation finding(s)?	◯	◯

18. What is the nature of the abnormal finding(s)?
- ☐ Crackles
- ☐ Wheezes
- ☐ Other (please specify below)

18a. Specify other __________________________________

19. Palpable lymph nodes?

	Yes	No
Cervical lymph nodes	◯	◯
Clavicular lymph nodes	◯	◯
Axillary lymph nodes	◯	◯

20. Oral lesions?
- ◯ Yes
- ◯ No

21. Skin lesions?
- ◯ Yes
- ◯ No

21a. What are the location(s) of the skin lesions? ____

22. Splenomegaly?
- ◯ Yes
- ◯ No
- ◯ Unable to palpate

23. Hepatomegaly?

◯ Yes
◯ No
◯ Unable to palpate

24. Are there are any other important findings on general examination?

____

Please read the following statement to the study participant at the end of the clinical examination form:
“This is the end of the clinical examination form. We will now collect a blood sample.”

E. FORM COMPLETION

25. End time:

____

26. ID of attending research team member?

____

Specimen Collection

A. Specimen Collection

1. Attending research team member ID

____

2. Date

____

3. Start time

____

4. Blood sample collected?

○ Yes
○ No

5. EDTA tube sample code (format: X-XXX-WB)

____

6. Plain tube sample code (format: X-XXX-S)

____

If any adverse events are observed, please complete an Adverse events reporting form and upload image of completed form.

B. FORM COMPLETION

7. End time

____

8. Attending research team member ID

____

Form upload

A. UPLOAD FILE

1. Date ______________________

2. Start time ______________________

3. Upload file type
- ☐ Adverse events reporting form
- ☐ Study Participant withdrawal form

4. Adverse events reporting form

5. Study participant withdrawal form

B. FORM COMPLETION

6. End Time ______________________

7. ID of attending research team member who completed this form ______________________
