# Supplementary material for: Histoplasma seropositivity and environmental risk factors for exposure in a general population in Upper River Region, The Gambia: A cross-sectional study
Source: One Health. 2024 Mar 27;18:100717. doi: 10.1016/j.onehlt.2024.100717 (PMC10992707; doi:10.1016/j.onehlt.2024.100717)
Supplement: Supplementary Table S1 — Univariable logistic regression analysis results, examining associations between Histoplasma seropositivity based on LAT result and demographic variables, amongst study participants (n = 298) in Upper River Region, The Gambia. Frequencies (n), percentages (%), Odds Ratios (OR), 95% Confidence Intervals (CIs) and p-values, were calculated using IBM SPSS Statistics 27. [file mmc3.docx]

**S1 Table.** Univariable logistic regression analysis results, examining associations between *Histoplasma* seropositivity based on LAT result and demographic variables, amongst study participants (*n*=298) in Upper River Region, The Gambia. Frequencies (*n*), percentages (%), Odds Ratios (OR), 95% Confidence Intervals (CIs) and *p*-values, were calculated using IBM SPSS Statistics 27.

| Variable | Frequency, n (%), total N=298 | *Histoplasma* seropositive, n (%), total N=56 | *Histoplasma* seronegative, n (%), total N=242 | Odds Ratio (95% CI) | *p-*value |
| --- | --- | --- | --- | --- | --- |
| Demographic | | | | | |
| Gender |  |  |  |  |  |
| Male (ref) | 133 (44·6) | 20 (15·0) | 113 (85·0) | 1·00 |  |
| Female | 165 (55·4) | 36 (21·8) | 129 (78·2) | 0·63 (0·35-1·16) | 0·14** |
| Age, years |  |  |  |  |  |
| Median (IQR) | 27·0 (15·8-42·0) | - | - | 0·97 (0·95-0·99) | 0·001* |
| Ethnic group |  |  |  |  |  |
| Mandinka (ref) | 102 (34·2) | 18 (17·6) | 84 (82·4) | 1·00 |  |
| Serahule | 89 (29·9) | 20 (22·5) | 69 (77·5) | 1·35 (0·66-2·76) | 0·41 |
| Wolof | 1 (0·3) | 0 (0·0) | 1 (100·0) | 0·00 (0·00-) | 1·00 |
| Fula | 100 (33·6) | 17 (17·0) | 83 (83·0) | 0·96 (0·46-1·98) | 0·90 |
| Mende | 1 (0·3) | 0 (0·0) | 1 (100·0) | 0·00 (0·00-) | 1·00 |
| No response | 5 (1·7) | 1 (20·0) | 4 (80·0) | 1·17 (0·12-11·07) | 0·89 |
| Occupational |  |  |  |  |  |
| Primary occupation(s) or household role(s) | | | | | |
| Farming |  |  |  |  |  |
| No (ref) | 108 (36·2) | 21 (19·4) | 87 (80·6) | 1·00 |  |
| Yes | 185 (62·1) | 34 (18·4) | 151 (81·6) | 0·93 (0·51-1·71) | 0·82 |
| No response | 5 (1·7) | 1 (20·0) | 4 (80·0) | 1·04 (0·11-9·75) | 0·98 |
| Business |  |  |  |  |  |
| No (ref) | 266 (89·3) | 53 (19·9) | 213 (80·1) | 1·00 |  |
| Yes | 27 (9·1) | 2 (7·4) | 25 (92·6) | 0·32 (0·07-1·40) | 0·13** |
| No response | 5 (1·7) | 1 (20·0) | 4 (80·0) | 1·01 (0·11-9·18) | 1·00 |
| Domestic work |  |  |  |  |  |
| No (ref) | 111 (37·1) | 12 (10·8) | 99 (89·2) | 1·00 |  |
| Yes | 182 (61·1) | 43 (23·6) | 139 (76·4) | 2·55 (1·28-5·09) | 0·008* |
| No response | 5 (1·7) | 1 (20·0) | 4 (80·0) | 2·06 (0·21-20·00) | 0·53 |
| Student |  |  |  |  |  |
| No (ref) | 211 (70·8) | 32 (15·2) | 179 (84·8) | 1·00 |  |
| Yes | 82 (27·5) | 23 (28·0) | 59 (72·0) | 2·18 (1·18-4·02) | 0·01* |
| No response | 5 (1·7) | 1 (20·0) | 4 (80·0) | 1·40 (0·15-12·92) | 0·77 |
| Other |  |  |  |  |  |
| No (ref) | 265 (88·9) | 53 (20·0) | 212 (80·0) | 1·00 |  |
| Yes | 28 (9·4) | 2 (7·1) | 26 (92·9) | 0·31 (0·07-1·34) | 0·12** |
| No response | 5 (1·7) | 1 (20·0) | 4 (80·0) | 1·00 (0·11-9·13) | 1·00 |
| No occupation or household role |  |  |  |  |  |
| No (ref) | 289 (97·0) | 55 (19·0) | 234 (81·0) | 1·00 |  |
| Yes | 4 (1·3) | 0 (0·0) | 4 (100·0) | 0·00 (0·00-) | 1·00 |
| No response | 5 (1·7) | 1 (20·0) | 4 (80·0) | 1·06 (0·12-9·71) | 0·96 |
| Soil excavation ^a^ |  |  |  |  |  |
| No (ref) | 78 (26·2) | 12 (15·4) | 66 (84·6) | 1·00 |  |
| Yes | 215 (72·1) | 43 (20·0) | 172 (80·0) | 1·38 (0·68-2·77) | 0·37 |
| No response | 5 (1·7) | 1 (20·0) | 4 (80·0) | 1·38 (0·14-13·39) | 0·78 |
| Building or construction |  |  |  |  |  |
| No (ref) | 271 (90·9) | 52 (19·2) | 219 (80·8) | 1·00 |  |
| Yes | 22 (7·4) | 3 (13·6) | 19 (86·4) | 0·67 (0·19-2·33) | 0·52 |
| No response | 5 (1·7) | 1 (20·0) | 4 (80·0) | 1·05 (0·12-9·62) | 0·96 |
| Location |  |  |  |  |  |
| Enumeration Area ^b^ |  |  |  |  |  |
| EA1^R^ | 25 (8·4) | 2 (8·0) | 23 (92·0) | 2·09 (0·18-24·62) | 0·56 |
| EA2^R^ | 25 (8·4) | 2 (8·0) | 23 (92·0) | 2·09 (0·18-24·62) | 0·56 |
| EA3^U^ (ref) | 25 (8·4) | 1 (4·0) | 24 (96·0) | 1·00 |  |
| EA4^U^ | 25 (8·4) | 5 (20·0) | 20 (80·0) | 6·00 (0·65-55·66) | 0·12** |
| EA5^R^ | 24 (8·1) | 6 (25·0) | 18 (75·0) | 8·00 (0·88-72·45) | 0·06** |
| EA6^R^ | 25 (8·4) | 11 (44·0) | 14 (56·0) | 18·86 (2·20-161·99) | 0·007* |
| EA7^R^ | 25 (8·4) | 6 (24·0) | 19 (76·0) | 7·58 (0·84-68·46) | 0·07** |
| EA8^R^ | 25 (8·4) | 8 (32·0) | 17 (68·0) | 11·29 (1·29-98·89) | 0·03* |
| EA9^R^ | 25 (8·4) | 4 (16·0) | 21 (84·0) | 4·57 (0·47-44·17) | 0·19** |
| EA10^U^ | 25 (8·4) | 0 (0·0) | 25 (100·0) | 0·00 (0·00-) | 1·00 |
| EA11^R^ | 24 (8·1) | 4 (16·7) | 20 (83·3) | 4·80 (0·59-0-46·47) | 0·18** |
| EA12^R^ | 25 (8·4) | 7 (28·0) | 18 (72·0) | 9·33 (1·05-82·78) | 0·05* |
| Settlement category |  |  |  |  |  |
| Urban | 75 (25·1) | 6 (8·0) | 69 (92·0) | 1·00 |  |
| Rural | 224 (74·9) | 50 (22·4) | 173 (77·6) | 3·32 (1·36-8·11) | 0·008* |
| Ward ^b^ |  |  |  |  |  |
| W1 (ref) | 49 (16·4) | 4 (8·2) | 45 (91·8) | 1·00 |  |
| W2 | 50 (16·8) | 6 (12·0) | 44 (88·0) | 1.53 (0·41-5·81) | 0·53 |
| W3 | 25 (8·4) | 11 (44·0) | 14 (56·0) | 8·84 (2·43-32·18) | <0·001* |
| W4 | 25 (8·4) | 7 (28·0) | 18 (72·0) | 4·38 (1·14-16·79) | 0·03* |
| W5 | 26 (8·7) | 2 (7·7) | 24 (92·3) | 0·94 (0·16-5·49) | 0·94 |
| W6 | 24 (8·1) | 6 (25·0) | 18 (75·0) | 3·75 (0·95-14·88) | 0·06** |
| W7 | 25 (8·4) | 2 (8·0) | 23 (92·0) | 0·98 (0·17-5·74) | 0·98 |
| W8 | 25 (8·4) | 8 (32·0) | 17 (68·0) | 5·29 (1·41-19·89) | 0·01* |
| W9 | 24 (8·1) | 6 (25·0) | 18 (75·0) | 3·75 (0·95-14·88) | 0·06** |
| W10 | 25 (8·4) | 4 (16·0) | 21 (84·0) | 2·14 (0·49-9·41) | 0·31 |
| District ^b^ |  |  |  |  |  |
| D1 (ref) | 74 (24·8) | 12 (16·2) | 62 (83·8) | 1·00 |  |
| D2 | 50 (16·8) | 4 (8·0) | 46 (92·0) | 0·45 (0·14-1·48) | 0·19** |
| D3 | 50 (16·8) | 14 (28·0) | 36 (72·0) | 2·01 (0·84-4·81) | 0·12** |
| D4 | 25 (8·4) | 7 (28·0) | 18 (72·0) | 2·01 (0·69-5·86) | 0·20 |
| D5 | 25 (8·4) | 11 (44·0) | 14 (56·0) | 4·06 (1·49-11·07) | 0·006* |
| D6 | 49 (16·4) | 4 (8·2) | 45 (91·8) | 0·46 (0·14-1·52) | 0·20 |
| D7 | 25 (8·4) | 4 (16·0) | 21 (84·0) | 0·98 (0·29-3·38) | 0·98 |

* *p*<0·05 (statistically significant), ** *p*<0·20; ^U^ Urban and ^R^ rural classification as per Gambian Bureau of Statistics (GBOS) 2013 Population and Housing Census, Economic Characteristics;^29^ ^a^ Encompasses all occupations involving soil excavation, including farming and construction; ^b^ Geographic divisions anonymised.
