# Supplementary material for: Histoplasma seropositivity and environmental risk factors for exposure in a general population in Upper River Region, The Gambia: A cross-sectional study
Source: One Health. 2024 Mar 27;18:100717. doi: 10.1016/j.onehlt.2024.100717 (PMC10992707; doi:10.1016/j.onehlt.2024.100717)
Supplement: Supplementary Table S2 — Univariable logistic regression analysis results, examining associations between Histoplasma seropositivity based on LAT result and environmental variables, amongst study participants (n = 298) in Upper River Region, The Gambia. Frequencies (n), percentages (%), Odds Ratios (OR), 95% Confidence Intervals (CIs) and p-values, were calculated using IBM SPSS Statistics 27. [file mmc4.docx]

**S2 Table.** Univariable logistic regression analysis results, examining associations between *Histoplasma* seropositivity based on LAT result and environmental variables, amongst study participants (*n*=298) in Upper River Region, The Gambia. Frequencies (*n*), percentages (%), Odds Ratios (OR), 95% Confidence Intervals (CIs) and *p*-values, were calculated using IBM SPSS Statistics 27.

| Variable | Frequency, n (%), total N=298 | *Histoplasma* seropositive, n (%), total N=56 | *Histoplasma* seronegative, n (%), total N=242 | Odds Ratio (95% CI) | *p-*value |
| --- | --- | --- | --- | --- | --- |
| Occupants per household | | | | | |
| Total (≥5 years), *n* |  |  |  |  |  |
| Median (IQR) | 22.0 (12.0-31.5) | - | - | 1.02 (1.00-1.05) | 0.05** |
| Children (5-17 years), *n* |  |  |  |  |  |
| Median (IQR) | 12.0 (6.0-20.0) | - | - | 1.01 (0.96-1.07) | 0.64 |
| Adults (≥18 years), *n* |  |  |  |  |  |
| Median (IQR) | 10.0 (6.0-10.5) | - | - | 1.04 (0.96-1.13) | 0.33 |
| Building materials^a^ | | | | | |
| Floors |  |  |  |  |  |
| Earth | 15 (5.0) | 1 (6.7) | 14 (93.3) | 0.28 (0.04-2.19) | 0.23 |
| Compacted mud | 8 (2.7) | 0 (0.0) | 8 (100.0) | 0.00 (0.00-) | 1.00 |
| Cement (ref) | 267 (89.6) | 54 (20.2) | 213 (79.8) | 1.00 |  |
| Tiles | 8 (2.7) | 1 (12.5) | 7 (87.5) | 0.56 (0.07-4.68) | 0.60 |
| Walls |  |  |  |  |  |
| Mud | 43 (14.4) | 2 (4.7) | 41 (95.3) | 0.18 (0.04-0.78) | 0.02* |
| Cement (ref) | 255 (85.6) | 54 (21.2) | 201 (78.8) | 1.00 |  |
| Roofs |  |  |  |  |  |
| (Corrugated) iron (ref) | 261 (87.6) | 54 (20.7) | 207 (79.3) | 1.00 |  |
| Thatch/ grass | 28 (9.4) | 2 (7.1) | 26 (92.9) | 0.30 (0.07-1.28) | 0.10** |
| Cement | 9 (3.0) | 0 (0.0) | 9 (100.0) | 0.00 (0.00-) | 1.00 |
| Compound boundary | | | | | |
| Boundary or fence |  |  |  |  |  |
| No | 6 (2.0) | 1 (16.7) | 5 (83.3) | 0.86 (0.10-7.53) | 0.89 |
| Yes (ref) | 292 (98.0) | 55 (18.8) | 237 (81.2) | 1.00 |  |
| Boundary fence material |  |  |  |  |  |
| Concrete (ref) | 205 (68.8) | 45 (22.0) | 160 (78.0) | 1.00 |  |
| Plants and iron | 5 (1.7) | 0 (0.0) | 5 (100.0) | 0.00 (0.00-) | 1.00 |
| Iron | 21 (7.0) | 6 (28.6) | 15 (71.4) | 1.42 (0.52-3.88) | 0.49 |
| Wood | 14 (4.7) | 1 (7.1) | 13 (92.9) | 0.27 (0.04-2.15) | 0.22 |
| Plants | 40 (13.4) | 2 (5.0) | 38 (95.0) | 0.19 (0.04-0.81) | 0.02* |
| NA/ no boundary fence | 6 (2.0) | 1 (16.7) | 5 (83.3) | 0.71 (0.08-6.24) | 0.76 |
| No response | 7 (2.3) | 1 (14.3) | 6 (85.7) | 0.59 (0.07-5.05) | 0.63 |
| Boundary fence opening |  |  |  |  |  |
| No | 9 (3.0) | 4 (44.4) | 5 (55.6) | 3.69 (0.96-14.24) | 0.06** |
| Yes (ref) | 275 (92.3) | 49 (17.8) | 226 (82.2) | 1.00 |  |
| NA/ No compound boundary | 6 (2.0) | 1 (16.7) | 5 (83.3) | 0.92 (0.11-8.07) | 0.94 |
| No response | 8 (2.7) | 2 (25.0) | 6 (75.0) | 1.54 (0.30-7.85) | 0.61 |
| Time boundary fence open |  |  |  |  |  |
| During day (ref) | 234 (78.5) | 41 (17.5) | 193 (82.5) | 1.00 |  |
| During day and night | 41 (13.8) | 8 (19.5) | 33 (80.5) | 1.14 (0.49-2.65) | 0.76 |
| NA/ No boundary opening | 9 (3.0) | 4 (44.4) | 5 (55.6) | 3.77 (0.97-14.63) | 0.06** |
| NA/ No boundary | 6 (2.0) | 1 (16.7) | 5 (83.3) | 0.94 (0.11-8.27) | 0.96 |
| No response | 8 (2.7) | 2 (25.0) | 6 (75.0) | 1.57 (0.31-8.05) | 0.59 |
| Ventilation and cooking | | | | | |
| Ventilation method |  |  |  |  |  |
| No ventilation system | 12 (4.0) | 1 (8.3) | 11 (91.7) | 0.38 (0.05-3.02) | 0.36 |
| Direct opening in wall or roof (+/- stand fan) (ref) | 286 (96.0) | 55 (19.2) | 231 (80.8) | 1.00 |  |
| Cooking location |  |  |  |  |  |
| Outside building(s)^a^ (ref) | 275 (92.3) | 53 (19.3) | 222 (80.7) | 1.00 |  |
| Inside building(s)^a^ | 15 (5.0) | 2 (13.3) | 13 (86.7) | 0.64 (0.14-2.94) | 0.57 |
| No response | 8 (2.7) | 1 (12.5) | 7 (87.5) | 0.60 (0.07-4.97) | 0.63 |
| Cooking fuel |  |  |  |  |  |
| Firewood (ref) | 293 (98.3) | 56 (19.1) | 237 (80.9) | 1.00 |  |
| Charcoal | 5 (1.7) | 0 (0.0) | 5 (100.0) | 0.00 (0.00-) | 1.00 |
| Water source | | | | | |
| Dry season |  |  |  |  |  |
| Borehole^b^ (ref) | 153 (51.3) | 34 (22.2) | 119 (77.8) | 1.00 |  |
| Covered well^b^ | 33 (11.1) | 9 (27.3) | 24 (72.7) | 1.31 (0.56-3.09) | 0.53 |
| Uncovered well^b^ | 13 (4.4) | 1 (7.7) | 12 (92.3) | 0.29 (0.04-2.32) | 0.25 |
| River | 8 (2.7) | 0 (0.0) | 8 (100.0) | 0.00 (0.00-) | 1.00 |
| Piped | 58 (19.5) | 12 (20.7) | 46 (79.3) | 0.91 (0.44-1.92) | 0.81 |
| Uncovered well^b^ and river | 5 (1.7) | 0 (0.0) | 5 (100.0) | 0.00 (0.00-) | 1.00 |
| Piped and borehole^b^ | 23 (7.7) | 0 (0.0) | 23 (100.0) | 0.00 (0.00-) | 1.00 |
| No response | 5 (1.7) | 0 (0.0) | 5 (100.0) | 0.00 (0.00-) | 1.00 |
| Rainy season |  |  |  |  |  |
| Borehole^b^ (ref) | 153 (51.3) | 34 (22.2) | 119 (77.8) | 1.00 |  |
| Covered well^b^ | 33 (11.1) | 9 (27.3) | 24 (72.7) | 1.31 (0.56-3.09) | 0.53 |
| Uncovered well^b^ | 13 (4.4) | 1 (7.7) | 12 (92.3) | 0.29 (0.04-2.32) | 0.25 |
| River | 13 (4.4) | 0 (0.0) | 13 (100.0) | 0.00 (0.00-) | 1.00 |
| Piped | 51 (17.1) | 12 (23.5) | 39 (76.5) | 1.08 (0.51-2.29) | 0.85 |
| Uncovered well^b^ and river | 0 (0.0) | 0 (0.0) | 0 (0.0) | - | - |
| Roof capture | 12 (4.0) | 0 (0.0) | 12 (100.0) | 0.00 (0.00-) | 1.00 |
| Piped and borehole^b^ | 23 (7.7) | 0 (0.0) | 23 (100.0) | 0.00 (0.00-) | 1.00 |
| Water treatment |  |  |  |  |  |
| No (ref) | 273 (91.6) | 54 (19.8) | 219 (80.2) | 1.00 |  |
| Yes | 25 (8.4) | 2 (8.0) | 23 (92.) | 0.35 (0.08-1.54) | 0.17** |
| Water treatment method |  |  |  |  |  |
| No treatment (ref) | 273 (91.6) | 54 (19.8) | 219 (80.2) | 1.00 |  |
| Filter | 25 (8.4) | 2 (8.0) | 23 (92.0) | 0.35 (0.08-1.54) | 0.17** |

* *p*<0.05 (statistically significant), ** *p*<0.20; ^a^ Building(s) where household occupants live; ^b^ Inside or outside compound boundary; NA=Not applicable.
