# Supplementary material for: Histoplasma seropositivity and environmental risk factors for exposure in a general population in Upper River Region, The Gambia: A cross-sectional study
Source: One Health. 2024 Mar 27;18:100717. doi: 10.1016/j.onehlt.2024.100717 (PMC10992707; doi:10.1016/j.onehlt.2024.100717)
Supplement: Supplementary Table S3 — Univariable logistic regression analysis results, examining associations between Histoplasma seropositivity based on LAT result and animal contact and management variables, amongst study participants (n = 298) in Upper River Region, The Gambia. Frequencies (n), percentages (%), Odds Ratios (OR), 95% Confidence Intervals (CIs) and p-values, were calculated using IBM SPSS Statistics 27. [file mmc5.docx]

**S3 Table.** Univariable logistic regression analysis results, examining associations between *Histoplasma* seropositivity based on LAT result and animal contact and management variables, amongst study participants (*n*=298) in Upper River Region, The Gambia. Frequencies (*n*), percentages (%), Odds Ratios (OR), 95% Confidence Intervals (CIs) and *p*-values, were calculated using IBM SPSS Statistics 27.

| Variable | Frequency, n (%), total N=298 | *Histoplasma* seropositive, n (%), total N=56 | *Histoplasma* seronegative, n (%), total N=242 | Odds Ratio (95% CI) | *p-*value |
| --- | --- | --- | --- | --- | --- |
| Domestic animal or livestock contact | | | | | |
| Domestic animal contact |  |  |  |  |  |
| No (ref) | 37 (12.4) | 2 (5.4) | 35 (94.6) | 1.00 |  |
| Yes | 256 (85.9) | 52 (20.3) | 204 (79.7) | 4.46 (1.04-19.15) | 0.04* |
| No response | 5 (1.7) | 2 (40.0) | 3 (60.0) | 11.67 (1.19-114.90) | 0.04* |
| Animal movement around compound |  |  |  |  |  |
| No compound boundary/ Enter compound freely (ref) | 203 (68.1) | 35 (17.2) | 168 (82.8) | 1.00 |  |
| Gate/ fence/ door in compound boundary | 64 (21.5) | 12 (18.8) | 52 (81.3) | 1.11 (0.54-2.29) | 0.78 |
| Animals tethered | 8 (2.7) | 2 (25.0) | 6 (75.0) | 1.60 (0.31-8.26) | 0.58 |
| NA/ No opening in fence | 15 (5.0) | 5 (33.3) | 10 (66.7) | 2.40 (0.77-7.46) | 0.13** |
| No response | 8 (2.7) | 2 (25.0) | 6 (75.0) | 1.60 (0.31-8.26) | 0.58 |
| Poultry contact ^a^ |  |  |  |  |  |
| No (ref) | 161 (54.0) | 27 (16.8) | 134 (83.2) | 1.00 |  |
| Yes | 132 (44.3) | 27 (20.5) | 105 (79.5) | 1.28 (0.71-2.31) | 0.42 |
| No response | 5 (1.7) | 2 (40.0) | 3 (60.0) | 3.31 (0.53-20.76) | 0.20 |
| Cattle contact ^a^ |  |  |  |  |  |
| No (ref) | 255 (85.6) | 43 (16.9) | 212 (83.1) | 1.00 |  |
| Yes | 38 (12.8) | 11 (28.9) | 27 (71.1) | 2.01 (0.93-4.36) | 0.08** |
| No response | 5 (1.7) | 2 (40.0) | 3 (60.0) | 3.29 (0.53-20.27) | 0.20 |
| Equid contact ^a^ |  |  |  |  |  |
| No (ref) | 107 (35.9) | 13 (12.1) | 94 (87.9) | 1.00 |  |
| Yes | 186 (62.4) | 41 (22.0) | 145 (78.0) | 2.05 (1.04-4.02) | 0.04* |
| No response | 5 (1.7) | 2 (40.0) | 3 (60.0) | 4.82 (0.74-31.62) | 0.10** |
| Sheep or goat contact ^a^ |  |  |  |  |  |
| No (ref) | 68 (22.8) | 10 (14.7) | 58 (85.3) | 1.00 |  |
| Yes | 225 (75.5) | 44 (19.6) | 181 (80.4) | 1.41 (0.67-2.98) | 0.37 |
| No response | 5 (1.7) | 2 (40.0) | 3 (60.0) | 3.87 (0.57-26.14) | 0.17** |
| Dog contact ^a^ |  |  |  |  |  |
| No (ref) | 259 (86.9) | 48 (18.5)) | 211 (81.5) | 1.00 |  |
| Yes | 34 (11.4) | 6 (17.6) | 28 (82.4) | 0.94 (0.37-2.40) | 0.90 |
| No response | 5 (1.7) | 2 (40.0) | 3 (60.0) | 2.93 (0.48-18.02) | 0.25 |
| Cat contact ^a^ |  |  |  |  |  |
| No (ref) | 259 (86.9) | 47 (18.1) | 212 (81.9) | 1.00 |  |
| Yes | 34 (11.4) | 7 (20.6) | 27 (79.4) | 1.17 (0.48-2.85) | 0.73 |
| No response | 5 (1.7) | 2 (40.0) | 3 (60.0) | 3.01 (0.49-18.50) | 0.24 |
| Working equid ownership | | | | | |
| Horses owned, *n* ^b^ |  |  |  |  |  |
| Median (IQR) | 0.0 (0.0-2.5) | - | - | 1.08 (0.98-1.20) | 0.14** |
| Horse ownership |  |  |  |  |  |
| No horses owned (ref) | 194 (65.1) | 27 (13.9) | 167 (86.1) | 1.00 |  |
| Farming | 96 (32.2) | 27 (28.1) | 69 (71.9) | 2.42 (1.33-4.42) | 0.004* |
| Farming and transport | 8 (2.7) | 2 (25.0) | 6 (75.0) | 2.06 (0.40-10.75) | 0.39 |
| Donkeys owned, *n* ^b^ |  |  |  |  |  |
| Median (IQR) | 2.0 (1.0-4.0) | - | - | 1.15 (1.03-1.29) | 0.01* |
| Donkey ownership |  |  |  |  |  |
| No donkeys owned (ref) | 47 (15.8) | 2 (4.3) | 45 (95.7) | 1.00 |  |
| Transport | 55 (18.5) | 20 (36.4) | 35 (63.6) | 12.86 (2.81-58.74) | <0.001* |
| Farming and transport | 188 (63.1) | 33 (17.6) | 155 (82.4) | 4.79 (1.11-20.74) | 0.04* |
| No response | 8 (2.7) | 1 (12.5) | 7 (87.5) | 3.21 (0.26-40.31) | 0.37 |
| Mules owned, *n* ^b^ |  |  |  |  |  |
| Median (IQR) | 0.0 (0.0-0.0) | - | - | 1.86 (0.99-3.50) | 0.05** |
| Mule ownership |  |  |  |  |  |
| No mules owned (ref) | 281 (94.3) | 49 (17.4) | 232 (82.6) | 1.00 |  |
| Farming | 17 (5.7) | 7 (41.2) | 10 (58.8) | 3.31 (1.20-9.14) | 0.02* |
| Domestic animal or livestock management in relation to compound | | | | | |
| Animal access to compound building(s) |  |  |  |  |  |
| No (ref) | 285 (95.6) | 55 (19.3) | 230 (80.7) | 1.00 |  |
| Yes | 13 (4.4) | 1 (7.7) | 12 (92.3) | 0.35 (0.04-2.74) | 0.32 |
| Animal management (1): Rainy season, day |  |  |  |  |  |
| Free compound entry/ exit (ref) | 32 (10.7) | 3 (9.4) | 29 (90.6) | 1.00 |  |
| Fenced area outside | 31 (10.4) | 4 (12.9) | 27 (87.1) | 1.43 (0.29-7.00) | 0.66 |
| Fenced area inside | 43 (14.4) | 9 (20.9) | 34 (79.1) | 2.56 (0.63-10.35) | 0.19** |
| Tethered outside | 151 (50.7) | 30 (19.9) | 121 (80.1) | 2.40 (0.68-8.40) | 0.17** |
| Tethered inside | 33 (11.1) | 10 (30.3) | 23 (69.7) | 4.20 (1.04-17.07) | 0.045* |
| Other ^c^ | 8 (2.7) | 0 (0.0) | 8 (100.0) | 0.00 (0.00-) | 1.00 |
| Animal management (2): Rainy season, day |  |  |  |  |  |
| Free compound entry/ exit (ref) | 32 (10.7) | 3 (9.4) | 29 (90.6) | 1.00 |  |
| Fenced/ tethered/ housed outside | 182 (61.1) | 34 (18.7) | 148 (81.3) | 2.22 (0.64-7.72) | 0.21 |
| Fenced/ tethered/ housed inside | 76 (25.5) | 19 (25.0) | 57 (75.0) | 3.22 (0.88-11.79) | 0.08** |
| Other ^c^ | 8 (2.7) | 0 (0.0) | 8 (100.0) | 0.00 (0.00-) | 1.00 |
| Animal management (1): Rainy season, night |  |  |  |  |  |
| Free compound entry/ exit (ref) | 25 (8.4) | 2 (8.0) | 23 (92.0) | 1.00 |  |
| Fenced area outside | 23 (7.7) | 4 (17.4) | 19 (82.6) | 2.42 (0.40-14.69) | 0.34 |
| Fenced area inside | 86 (28.9) | 25 (29.1) | 61 (70.9) | 4.71 (1.03-21.51) | 0.045* |
| Tethered outside | 45 (15.1) | 6 (13.3) | 39 (86.7) | 1.77 (0.33-9.50) | 0.51 |
| Tethered inside | 111 (37.2) | 19 (17.1) | 92 (82.9) | 2.38 (0.52-10.94) | 0.27 |
| Housed area outside | 8 (2.7) | 0 (0.0) | 8 (100.0) | 0.00 (0.00-) | 1.00 |
| Animal management (2): Rainy season, night |  |  |  |  |  |
| Free compound entry/ exit (ref) | 25 (8.4) | 2 (8.0) | 23 (92.0) | 1.00 |  |
| Fenced/ tethered/ housed outside | 68 (22.8) | 10 (14.7) | 58 (85.3) | 1.98 (0.40-9.75) | 0.40 |
| Fenced/ tethered/ housed inside | 197 (66.1) | 44 (22.3) | 153 (77.7) | 3.31 (0.75-14.58) | 0.11** |
| Animal management (1): Dry season, day |  |  |  |  |  |
| Free compound entry/ exit (ref) | 143 (48.0) | 26 (18.2) | 117 (81.8) | 1.00 |  |
| Fenced area outside | 23 (7.7) | 4 (17.4) | 19 (82.6) | 0.95 (0.30-3.02) | 0.93 |
| Fenced area inside | 30 (10.1) | 7 (23.3) | 23 (76.7) | 1.37 (0.53-3.53) | 0.52 |
| Tethered outside | 15 (5.0) | 0 (0.0) | 15 (100.0) | 0.00 (0.00-) | 1.00 |
| Tethered inside | 79 (26.5) | 15 (19.0) | 64 (81.0) | 1.06 (0.52-2.13) | 0.88 |
| No response | 8 (2.7) | 4 (50.0) | 4 (50.0) | 4.50 (1.06-19.18) | 0.04* |
| Animal management (2): Dry season, day |  |  |  |  |  |
| Free compound entry/ exit (ref) | 143 (48.0) | 26 (18.2) | 117 (81.8) | 1.00 |  |
| Fenced/ tethered/ housed outside | 38 (12.8) | 4 (10.5) | 34 (89.5) | 0.53 (0.17-1.62) | 0.27 |
| Fenced/ tethered/ housed inside | 109 (36.6) | 22 (20.2) | 87 (79.8) | 1.14 (0.61-2.14) | 0.69 |
| No response | 8 (2.7) | 4 (50.0) | 4 (50.0) | 4.50 (1.06-19.18) | 0.04* |
| Animal management (1): Dry season, night |  |  |  |  |  |
| Free compound entry/ exit (ref) | 40 (13.4) | 2 (5.0) | 38 (95.0) | 1.00 |  |
| Fenced area outside | 44 (14.8) | 8 (18.2) | 36 (81.8) | 4.22 (0.84-21.23) | 0.08** |
| Fenced area inside | 73 (24.5) | 20 (27.4) | 53 (72.6) | 7.17 (1.58-32.52) | 0.01* |
| Tethered outside | 21 (7.0) | 3 (14.3) | 18 (85.7) | 3.17 (0.49-20.65) | 0.23 |
| Tethered inside | 120 (40.3) | 23 (19.2) | 97 (80.8) | 4.51 (1.01-20.05) | 0.048* |
| Animal management (2): Dry season, night |  |  |  |  |  |
| Free compound entry/ exit (ref) | 40 (13.4) | 2 (5.0) | 38 (95.0) | 1.00 |  |
| Fenced/ tethered/ housed outside | 65 (21.8) | 11 (16.9) | 54 (83.1) | 3.87 (0.81-18.47) | 0.09** |
| Fenced/ tethered/ housed inside | 193 (64.8) | 43 (22.3) | 150 (77.7) | 5.45 (1.26-23.50) | 0.02* |
| Domestic animal or livestock manure management (in previous 1 month) | | | | | |
| Animal manure clearing/ disposal |  |  |  |  |  |
| No (ref) | 82 (27.5) | 6 (7.3) | 76 (92.7) | 1.00 |  |
| Yes | 212 (71.1) | 49 (23.1) | 163 (76.9) | 3.81 (1.56-9.28) | 0.003* |
| No response | 4 (1.3) | 1 (25.0) | 3 (75.0) | 4.22 (0.38-47.06) | 0.24 |
| Animal manure clearing/ disposal – specify animal |  |  |  |  |  |
| No manure clearing (ref) | 81 (27.2) | 6 (7.4) | 75 (92.6) | 1.00 |  |
| Equids | 14 (4.7) | 4 (28.6) | 10 (71.4) | 5.00 (1.20-20.83) | 0.03* |
| Sheep or goats | 57 (19.1) | 7 (12.3) | 50 (87.7) | 1.75 (0.56-5.51) | 0.34 |
| Equids and sheep or goats | 128 (43.0) | 35 (27.3) | 93 (72.7) | 4.70 (1.88-11.78) | <0.001* |
| Cattle | 1 (0.3) | 0 (0.0) | 1 (100.0) | 0.00 (0.00-) | 1.00 |
| Other | 12 (4.0) | 3 (25.0) | 9 (75.0) | 4.17 (0.89-19.61) | 0.07** |
| No response | 5 (1.7) | 1 (20.0) | 4 (80.0) | 3.13 (0.30-32.56) | 0.34 |
| Animal manure collection ^d^ |  |  |  |  |  |
| No (ref) | 76 (25.5) | 5 (6.6) | 71 (93.4) | 1.00 |  |
| Yes | 217 (72.8) | 50 (23.0) | 167 (77.0) | 4.25 (1.63-11.11) | 0.003* |
| No response | 5 (1.7) | 1 (20.0) | 4 (80.0) | 3.55 (0.33-38.03) | 0.30 |
| Animal manure collection ^d^ – specify animal |  |  |  |  |  |
| No manure collection (ref) | 76 (25.5) | 5 (6.6) | 71 (93.4) | 1.00 |  |
| Equids | 17 (5.7) | 5 (29.4) | 12 (70.6) | 5.92 (1.49-23.57) | 0.01* |
| Sheep or goats | 64 (21.5) | 9 (14.1) | 55 (85.9) | 2.32 (0.74-7.33) | 0.15** |
| Equids and sheep or goats | 130 (43.6) | 34 (26.2) | 96 (73.8) | 5.03 (1.87-13.50) | 0.001* |
| Equids and poultry | 3 (1.0) | 1 (33.3) | 2 (66.7) | 7.10 (0.55-92.40) | 0.13** |
| Other | 4 (1.3) | 1 (25.0) | 3 (75.0) | 4.73 (0.41-54.20) | 0.21 |
| No response | 4 (1.3) | 1 (25.0) | 3 (75.0) | 4.73 (0.41-54.20) | 0.21 |
| Animal manure clearing/disposal or collection |  |  |  |  |  |
| No (ref) | 75 (25.2) | 5 (6.7) | 70 (93.3) | 1.00 |  |
| Yes | 218 (73.2) | 50 (22.9) | 168 (77.1) | 4.17 (1.59-10.89) | 0.004* |
| No response | 5 (1.7) | 1 (20.0) | 4 (80.0) | 3.50 (0.33-37.51) | 0.30 |
| Other domestic animal or livestock contact activities (in previous 1 month) | | | | | |
| Animal feeding or grazing |  |  |  |  |  |
| No (ref) | 137 (46.0) | 22 (16.1) | 115 (83.9) | 1.00 |  |
| Yes | 149 (50.0) | 32 (21.5) | 117 (78.5) | 1.43 (0.78-2.61) | 0.24 |
| No response | 12 (4.0) | 2 (16.7) | 10 (83.3) | 1.05 (0.21-5.10) | 0.96 |
| Animal feeding or grazing – specify animal |  |  |  |  |  |
| No feeding or grazing (ref) | 137 (46.0) | 22 (16.1) | 115 (83.9) | 1.00 |  |
| Equids | 63 (21.1) | 16 (25.4) | 47 (74.6) | 1.78 (0.86-3.69) | 0.12** |
| Sheep or goats | 56 (18.8) | 10 (17.9) | 46 (82.1) | 1.14 (0.50-2.59) | 0.76 |
| Equids and sheep or goats | 26 (8.7) | 5 (19.2) | 21 (80.8) | 1.25 (0.42-3.65) | 0.69 |
| Other | 4 (1.3) | 1 (25.0) | 3 (75.0) | 1.74 (0.17-17.53) | 0.64 |
| No response | 12 (4.0) | 2 (16.7) | 10 (83.3) | 1.05 (0.21-5.10) | 0.96 |
| Animal slaughter or skinning or butchering |  |  |  |  |  |
| No (ref) | 292 (98.0) | 55 (18.8) | 237 (81.2) | 1.00 |  |
| Yes | 2 (0.7) | 0 (0.0) | 2 (100.0) | 0.00 (0.00-) | 1.00 |
| No response | 4 (1.3) | 1 (25.0) | 3 (75.0) | 1.44 (0.15-14.07) | 0.76 |
| Burial of dead animals |  |  |  |  |  |
| No (ref) | 292 (98.0) | 54 (18.5) | 238 (81.5) | 1.00 |  |
| Yes | 6 (2.0) | 2 (33.3) | 4 (66.7) | 2.20 (0.39-12.34) | 0.37 |

* *p*<0.05 (statistically significant), ** *p*<0.20; ^a^ Describes direct contact with specified domestic animal species; ^b^ Median and Interquartile Range (IQR) statistics calculated at household-level; ^c^ Fenced area outside and tethered inside; ^d^ Describes involvement in collecting or storing animal manure in proximity to compound environment; NA=Not applicable.
