# Supplementary material for: Histoplasma seropositivity and environmental risk factors for exposure in a general population in Upper River Region, The Gambia: A cross-sectional study
Source: One Health. 2024 Mar 27;18:100717. doi: 10.1016/j.onehlt.2024.100717 (PMC10992707; doi:10.1016/j.onehlt.2024.100717)
Supplement: Supplementary Table S4 — Univariable logistic regression analysis results, examining associations between Histoplasma seropositivity based on LAT result and wildlife observation variables, amongst study participants (n = 298) in Upper River Region, The Gambia. Frequencies (n), percentages (%), Odds Ratios (OR), 95% Confidence Intervals (CIs) and p-values, were calculated using IBM SPSS Statistics 27. [file mmc6.docx]

**S4 Table.** Univariable logistic regression analysis results, examining associations between *Histoplasma* seropositivity based on LAT result and wildlife observation variables, amongst study participants (*n*=298) in Upper River Region, The Gambia. Frequencies (*n*), percentages (%), Odds Ratios (OR), 95% Confidence Intervals (CIs) and *p*-values, were calculated using IBM SPSS Statistics 27.

| Variable | Frequency, *n* (%), total *N*=298 | *Histoplasma* seropositive, *n* (%), total *N*=56 | *Histoplasma* seronegative, *n* (%), total *N*=242 | Odds Ratio (95% CI) | *p-*value |
| --- | --- | --- | --- | --- | --- |
| Wildlife observation (in previous 7 days) | | | | | |
| Wildlife observation |  |  |  |  |  |
| No (ref) | 25 (8.4) | 5 (20.0) | 20 (80.0) | 1.00 |  |
| Yes | 269 (90.3) | 50 (18.6) | 219 (81.4) | 0.91 (0.33-2.55) | 0.86 |
| No response | 4 (1.3) | 1 (25.0) | 3 (75.0) | 1.33 (0.11-15.70) | 0.82 |
| Bat observation |  |  |  |  |  |
| No (ref) | 274 (91.9) | 50 (18.2) | 224 (81.8) | 1.00 |  |
| Yes | 20 (6.7) | 5 (25.0) | 15 (75.0) | 1.49 (0.52-4.30) | 0.46 |
| No response | 4 (1.3) | 1 (25.0) | 3 (75.0) | 1.49 (0.15-14.66) | 0.73 |
| Wild bird observation |  |  |  |  |  |
| No (ref) | 290 (97.3) | 55 (19.0) | 235 (81.0) | 1.00 |  |
| Yes | 4 (1.3) | 0 (0.0) | 4 (100.0) | 0.00 (0.00-) | 1.00 |
| No response | 4 (1.3) | 1 (25.0) | 3 (75.0) | 1.42 (0.15-13.95) | 0.76 |
| Rat observation |  |  |  |  |  |
| No (ref) | 29 (9.7) | 6 (20.7) | 23 (79.3) | 1.00 |  |
| Yes | 265 (88.9) | 49 (18.5) | 216 (81.5) | 0.87 (0.34-2.25) | 0.77 |
| No response | 4 (1.3) | 1 (25.0) | 3 (75.0) | 1.28 (0.11-14.59) | 0.84 |
| Monkey observation |  |  |  |  |  |
| No (ref) | 291 (97.7) | 55 (18.9) | 236 (81.1) | 1.00 |  |
| Yes | 3 (1.0) | 0 (0.0) | 3 (100.0) | 0.00 (0.00-) | 1.00 |
| No response | 4 (1.3) | 1 (25.0) | 3 (75.0) | 1.43 (0.15-14.01) | 0.76 |
| Lizard observation |  |  |  |  |  |
| No (ref) | 28 (9.4) | 5 (17.9) | 23 (82.1) | 1.00 |  |
| Yes | 266 (89.3) | 50 (18.8) | 216 (81.2) | 1.07 (0.39-2.94) | 0.90 |
| No response | 4 (1.3) | 1 (25.0) | 3 (75.0) | 1.53 (0.13-17.97) | 0.73 |
| Wildlife faeces observed in compound |  |  |  |  |  |
| No (ref) | 60 (20.1) | 12 (20.0) | 48 (80.0) | 1.00 |  |
| Yes | 232 (77.9) | 42 (18.1) | 190 (81.9) | 0.88 (0.43-1.81) | 0.74 |
| No response | 6 (2.0) | 2 (33.3) | 4 (66.7) | 2.00 (0.33-12.24) | 0.45 |

* *p*<0.05 (statistically significant), ** *p*<0.20.
