# Supplementary material for: Histoplasma seropositivity and environmental risk factors for exposure in a general population in Upper River Region, The Gambia: A cross-sectional study
Source: One Health. 2024 Mar 27;18:100717. doi: 10.1016/j.onehlt.2024.100717 (PMC10992707; doi:10.1016/j.onehlt.2024.100717)
Supplement: Supplementary Table S6 — Baseline demographic characteristics of study sample and Basse LGA population, using the Gambia Bureau of Statistics (GBoS) 2022–3 population projection data and 2013 Population and Housing Census data. [file mmc8.docx]

**S6 Table.** Baseline demographic characteristics of study sample and Basse LGA population, using the Gambia Bureau of Statistics (GBOS) 2022-3 population projection data and 2013 Population and Housing Census data.

| Variable | Study population, *N*=298 | Basse LGA population |
| --- | --- | --- |
| GBOS Basse LGA population projection data 2022, total population *N*=298867 | | |
| Sex |  |  |
| Male | 133 (44.6) | 143456 (48.0) |
| Female | 165 (55.4) | 155411 (52.0) |
| GBOS Basse LGA population projection data 2023, population ≥5 years *N*=252954 | | |
| Age category, years |  |  |
| 5-9 | 26 (8.7) | 52043 (20.6) |
| 10-14 | 43 (14.4) | 39870 (15.8) |
| 15-19 | 41 (13.8) | 34627 (13.7) |
| 20-24 | 27 (9.1) | 26230 (10.4) |
| 25-29 | 28 (9.4) | 23824 (9.4) |
| 30-34 | 19 (6.4) | 17240 (6.8) |
| 35-39 | 28 (9.4) | 13271 (5.3) |
| 40-44 | 18 (6.0) | 10774 (4.3) |
| 45-49 | 13 (4.4) | 8134 (3.2) |
| 50-54 | 13 (4.4) | 6930 (2.7) |
| 55-59 | 10 (3.4) | 4080 (1.6) |
| 60-64 | 9 (3.0) | 5058 (2.0) |
| 65-69 | 12 (4.0) | 3077 (1.2) |
| 70-74 | 7 (2.3) | 2880 (1.1) |
| 75-79 | 2 (0.7) | 1657 (0.7) |
| 80-84 | 2 (0.7) | 1551 (0.6) |
| 85+ | 0 (0.0) | 1707 (0.7) |
| GBOS 2013 Population and Housing Census: Housing and Household Characteristics | | |
| Average household size |  |  |
| Total, mean (median) | 23.4 (22.0) | 14.9 ^a^ |
| Rural, mean (median) | 23.2 (22.5) | 17.0 ^a^ |
| Urban, mean (median) | 23.8 (22.0) | 11.5 ^a^ |

^a^ Median values not available (no access to raw data).
