# Supplementary material for: Histoplasma seropositivity and environmental risk factors for exposure in a general population in Upper River Region, The Gambia: A cross-sectional study
Source: One Health. 2024 Mar 27;18:100717. doi: 10.1016/j.onehlt.2024.100717 (PMC10992707; doi:10.1016/j.onehlt.2024.100717)
Supplement: Supplementary Table S7 — The frequency distribution of IMMY Latex Agglutination-Histoplasma test results for study participants (n = 298), categorised by reaction strength, and result interpretation. [file mmc9.docx]

**S7 Table.** The frequency distribution of IMMY Latex Agglutination-*Histoplasma* test results for study participants (*n*=298), categorised by reaction strength, and result interpretation.

|  | | **Description (IMMY, 2018)**^35^ | **Study participants, *n* (%)** |
| --- | --- | --- | --- |
| **LAT reaction strength** | - | A homogeneous suspension of particles with no visible clumping | 178 (59.7) |
|  | 1+ | Fine granulation against a milky background | 64 (21.5) |
|  | 2+ | Small but definite clumps against a slightly cloudy background | 47 (15.8) |
|  | 3+ | Large and small clumps against a clear background | 9 (3.0) |
|  | 4+ | Large clumps against a very clear background | 0 (0.0) |
| **LAT result interpretation** | Negative | Reaction strength – or 1+ | 242 (81.2) |
|  | Positive | Reaction strength 2+ to 4+ | 56 (18.8) |
