# Supplementary material for: Histoplasma seropositivity and environmental risk factors for exposure in a general population in Upper River Region, The Gambia: A cross-sectional study
Source: One Health. 2024 Mar 27;18:100717. doi: 10.1016/j.onehlt.2024.100717 (PMC10992707; doi:10.1016/j.onehlt.2024.100717)
Supplement: Supplementary Table S8 — Univariable logistic regression analysis results, examining associations between Histoplasma seropositivity based on LAT result and clinical variables, amongst study participants (n = 298) in Upper River Region, The Gambia. Frequencies (n), percentages (%), Odds Ratios (OR), 95% Confidence Intervals (CIs) and p-values, were calculated using IBM SPSS Statistics 27. [file mmc10.docx]

**S8 Table. Univariable logistic regression analysis results, examining associations between *Histoplasma* seropositivity based on LAT result and clinical variables, amongst study participants (*n*=298) in Upper River Region, The Gambia. Frequencies (*n*), percentages (%), Odds Ratios (OR), 95% Confidence Intervals (CIs) and *p*-values, were calculated using IBM SPSS Statistics 27.**

| Variable | Frequency, n (%), total N=298 | *Histoplasma* seropositive, n (%), total N=56 | *Histoplasma* seronegative, n (%), total N=242 | Odds Ratio (95% CI) | *p-*value |
| --- | --- | --- | --- | --- | --- |
| Clinical history (reported by participant) | | | | | |
| Cough |  |  |  |  |  |
| No (ref) | 239 (80.2) | 47 (19.7) | 192 (80.3) | 1.00 |  |
| Yes | 59 (19.8) | 9 (15.3) | 50 (84.7) | 0.74 (0.34-1.60) | 0.44 |
| Cough type |  |  |  |  |  |
| Without mucus | 21 (7.0) | 4 (19.0) | 17 (81.0) | 0.96 (0.31-2.99) | 0.95 |
| With mucus | 19 (6.4) | 4 (21.1) | 15 (78.9) | 1.09 (0.35-3.43) | 0.88 |
| Mixed | 19 (6.4) | 1 (5.3) | 18 (94.7) | 0.23 (0.03-1.74) | 0.15** |
| No cough (ref) | 239 (80.2) | 47 (19.7) | 192 (80.3) | 1.00 |  |
| Shortness of breath |  |  |  |  |  |
| No (ref) | 292 (98.0) | 56 (19.2) | 236 (80.8) | 1.00 |  |
| Yes | 6 (2.0) | 0 (0.0) | 6 (100.0) | 0.00 (0.00-) | 1.00 |
| Chest pain |  |  |  |  |  |
| No (ref) | 280 (94.0) | 54 (19.3) | 226 (80.7) | 1.00 |  |
| Yes | 18 (6.0) | 2 (11.1) | 16 (88.9) | 0.52 (0.12-2.34) | 0.40 |
| Fever |  |  |  |  |  |
| No (ref) | 287 (96.3) | 53 (18.5) | 234 (81.5) | 1.00 |  |
| Yes | 11 (3.7) | 3 (27.3) | 8 (72.7) | 1.66 (0.43-6.45) | 0.47 |
| Night sweats |  |  |  |  |  |
| No (ref) | 297 (99.7) | 56 (18.9) | 241 (81.1) | 1.00 |  |
| Yes | 1 (0.3) | 0 (0.0) | 1 (100.0) | 0.00 (0.00-) | 1.00 |
| Loss of appetite |  |  |  |  |  |
| No (ref) | 291 (97.7) | 55 (18.9) | 236 (81.1) | 1.00 |  |
| Yes | 7 (2.3) | 1 (14.3) | 6 (85.7) | 0.72 (0.08-6.06) | 0.76 |
| Weight loss |  |  |  |  |  |
| No (ref) | 294 (98.7) | 55 (18.7) | 239 (81.3) | 1.00 |  |
| Yes | 4 (1.3) | 1 (25.0) | 3 (75.0) | 1.45 (0.15-14.19) | 0.75 |
| Skin lesions |  |  |  |  |  |
| No (ref) | 298 (100.0) | 56 (18.8) | 242 (81.2) | - |  |
| Yes | 0 (0.0) | 0 (0.0) | 0 (0.0) |  |  |
| Oral lesions |  |  |  |  |  |
| No (ref) | 297 (99.7) | 56 (18.9) | 241 (81.1) | 1.00 |  |
| Yes | 1 (0.3) | 0 (0.0) | 1 (100.0) | 0.00 (0.00-) | 1.00 |
| Myalgia |  |  |  |  |  |
| No (ref) | 285 (95.6) | 55 (19.3) | 230 (80.7) | 1.00 |  |
| Yes | 13 (4.4) | 1 (7.7) | 12 (92.3) | 0.35 (0.04-2.74) | 0.32 |
| Arthralgia |  |  |  |  |  |
| No (ref) | 277 (93.0) | 54 (19.5) | 223 (80.5) | 1.00 |  |
| Yes | 21 (7.0) | 2 (9.5) | 19 (90.5) | 0.44 (0.10-1.92) | 0.27 |
| Smoking status |  |  |  |  |  |
| Active smoker | 19 (6.4) | 2 (10.5) | 17 (89.5) | 0.46 (0.10-2.04) | 0.31 |
| Ex-smoker | 15 (5.0) | 0 (0.0) | 15 (100.0) | 0.00 (0.00-) | 1.00 |
| Never smoked (ref) | 264 (88.6) | 54 (20.5) | 210 (79.5) | 1.00 |  |
| Clinical examination findings | | | | | |
| Temperature, degrees Celsius |  |  |  |  |  |
| Median (IQR) | 36.5 (36.2-36.9) | - | - | 0.49 (0.23-1.03) | 0.06** |
| Respiratory rate, breaths per minute |  |  |  |  |  |
| Median (IQR) | 18.0 (17.0-19.0) | - | - | 1.12 (1.00-1.25) | 0.05** |
| Body Mass Index ^a^ |  |  |  |  |  |
| Median (IQR) | 20.0 (16.5-24.1) | - | - | 0.95 (0.90-1.01) | 0.08** |
| Chest pain |  |  |  |  |  |
| No (ref) | 281 (94.3) | 50 (17.8) | 231 (82.2) | 1.00 |  |
| Yes | 17 (5.7) | 6 (35.3) | 11 (64.7) | 2.52 (0.89-7.13) | 0.08** |
| Dyspnoea |  |  |  |  |  |
| No (ref) | 295 (99.0) | 56 (19.0) | 239 (81.0) | 1.00 |  |
| Yes | 3 (1.0) | 0 (0.0) | 3 (100.0) | 0.00 (0.00-) | 1.00 |
| Cough |  |  |  |  |  |
| No (ref) | 294 (98.7) | 56 (19.0) | 238 (81.0) | 1.00 |  |
| Yes | 4 (1.3) | 0 (0.0) | 4 (100.0) | 0.00 (0.00-) | 1.00 |
| Chest auscultation abnormal findings |  |  |  |  |  |
| No (ref) | 296 (99.3) | 56 (18.9) | 240 (81.1) | 1.00 |  |
| Yes | 2 (0.7) | 0 (0.0) | 2 (100.0) | 0.00 (0.00-) | 1.00 |
| Oral lesions |  |  |  |  |  |
| No (ref) | 296 (99.3) | 55 (18.6) | 241 (81.4) | 1.00 |  |
| Yes | 2 (0.7) | 1 (50.0) | 1 (50.0) | 4.38 (0.27-71.14) | 0.30 |
| Skin lesions |  |  |  |  |  |
| No (ref) | 295 (99.0) | 56 (19.0) | 239 (81.0) | 1.00 |  |
| Yes | 3 (1.0) | 0 (0.0) | 3 (100.0) | 0.00 (0.00-) | 1.00 |
| Palpable lymph nodes | | | | | |
| Cervical lymph nodes |  |  |  |  |  |
| No (ref) | 259 (86.9) | 50 (19.3) | 209 (80.7) | 1.00 |  |
| Yes | 39 (13.1) | 6 (15.4) | 33 (84.6) | 0.76 (0.30-1.91) | 0.56 |
| Clavicular lymph nodes |  |  |  |  |  |
| No (ref) | 258 (86.6) | 50 (19.4) | 208 (80.6) | 1.00 |  |
| Yes | 40 (13.4) | 6 (15.0) | 34 (85.0) | 0.73 (0.29-1.84) | 0.51 |
| Axillary lymph nodes |  |  |  |  |  |
| No (ref) | 259 (86.9) | 50 (19.3) | 209 (80.7) | 1.00 |  |
| Yes | 39 (13.1) | 6 (15.4) | 33 (84.6) | 0.76 (0.30-1.91) | 0.56 |
| Other findings on general examination | | | | | |
| None | 288 (96.6) | | | | |
| Hypertension | 1 (0.3) | | | | |
| Hypertension and diabetes | 2 (0.7) | | | | |
| Abdominal pain | 3 (1.0) | | | | |
| Cardiac problem | 1 (0.3) | | | | |
| Eye infection | 1 (0.3) | | | | |
| Ulcerative skin lesion | 1 (0.3) | | | | |
| No response | 1 (0.3) | | | | |
| Histoplasmosis awareness | | | | | |
| Histoplasmosis awareness prior to study |  |  |  |  |  |
| No (ref) | 255 (85.6) | 51 (20.0) | 204 (80.0) | 1.00 |  |
| Yes | 43 (14.4) | 5 (11.6) | 38 (88.4) | 0.53 (0.20-1.41) | 0.20 |
| Knowledge sources of histoplasmosis |  | | | | |
| None | 255 (85.6) | | | | |
| Research team | 8 (2.7) | | | | |
| Government healthcare worker | 2 (0.7) | | | | |
| Radio/ WhatsApp | 15 (5.0) | | | | |
| Government Livestock Agent | 14 (4.7) | | | | |
| Other ^b^ | 4 (1.3) | | | | |

* *p*<0.05 (statistically significant), ** *p*<0.20; ^a^ Erroneous height measurement entered for *n*=2 female participants, thus no BMI values for individuals contributed to statistical analyses; ^b^ Other: Word-of-mouth (*n*=2) and community meetings with non-governmental organisation (*n*=2).
