# Supplementary material for: Histoplasma seropositivity and environmental risk factors for exposure in a general population in Upper River Region, The Gambia: A cross-sectional study
Source: One Health. 2024 Mar 27;18:100717. doi: 10.1016/j.onehlt.2024.100717 (PMC10992707; doi:10.1016/j.onehlt.2024.100717)
Supplement: Supplementary Table S9 — Baseline demographic characteristics of study sample and Basse LGA population, using the Gambia Bureau of Statistics (GBOS) 2022–3 population projection data and 2013 Population and Housing Census data. [file mmc11.docx]

**S9 Table. Baseline demographic characteristics of study sample and Basse LGA population, using the Gambia Bureau of Statistics (GBOS) 2022-3 population projection data and 2013 Population and Housing Census data.**

| Variable | Study population, *N*=298 | Basse LGA population |
| --- | --- | --- |
| GBOS Basse LGA population projection data 2022, total population *N*=298867 | | |
| Sex |  |  |
| Male | 133 (44·6) | 143456 (48·0) |
| Female | 165 (55·4) | 155411 (52·0) |
| GBOS Basse LGA population projection data 2023, population ≥5 years *N*=252954 | | |
| Age category, years |  |  |
| 5-9 | 26 (8·7) | 52043 (20·6) |
| 10-14 | 43 (14·4) | 39870 (15·8) |
| 15-19 | 41 (13·8) | 34627 (13·7) |
| 20-24 | 27 (9·1) | 26230 (10·4) |
| 25-29 | 28 (9·4) | 23824 (9·4) |
| 30-34 | 19 (6·4) | 17240 (6·8) |
| 35-39 | 28 (9·4) | 13271 (5·3) |
| 40-44 | 18 (6·0) | 10774 (4·3) |
| 45-49 | 13 (4·4) | 8134 (3·2) |
| 50-54 | 13 (4·4) | 6930 (2·7) |
| 55-59 | 10 (3·4) | 4080 (1·6) |
| 60-64 | 9 (3·0) | 5058 (2·0) |
| 65-69 | 12 (4·0) | 3077 (1·2) |
| 70-74 | 7 (2·3) | 2880 (1·1) |
| 75-79 | 2 (0·7) | 1657 (0·7) |
| 80-84 | 2 (0·7) | 1551 (0·6) |
| 85+ | 0 (0.0) | 1707 (0·7) |
| GBOS 2013 Population and Housing Census: Housing and Household Characteristics | | |
| Average household size |  |  |
| Total, mean (median) | 23·4 (22·0) | 14·9 ^a^ |
| Rural, mean (median) | 23·2 (22·5) | 17·0 ^a^ |
| Urban, mean (median) | 23·8 (22·0) | 11·5 ^a^ |

^a^ Median values not available (no access to raw data).
